# Circulating amino acids and the risk of macrovascular, microvascular and mortality outcomes in individuals with type 2 diabetes: results from the ADVANCE trial

**DOI:** 10.1007/s00125-018-4619-x

**Published:** 2018-05-04

**Authors:** Paul Welsh, Naomi Rankin, Qiang Li, Patrick B. Mark, Peter Würtz, Mika Ala-Korpela, Michel Marre, Neil Poulter, Pavel Hamet, John Chalmers, Mark Woodward, Naveed Sattar

**Affiliations:** 1 BHF Glasgow Cardiovascular Research Centre, Institute of Cardiovascular & Medical Sciences, University of Glasgow, 126 University Place, Glasgow, G12 8TA UK; 2 The George Institute for Global Health, University of New South Wales, Sydney, NSW Australia; 3 Research Programs Unit, Diabetes and Obesity, University of Helsinki, Helsinki, Finland; 4 Nightingale Health Ltd, Helsinki, Finland; 5 Computational Medicine, Faculty of Medicine, University of Oulu and Biocenter Oulu, Oulu, Finland; 6 NMR Metabolomics Laboratory, School of Pharmacy, University of Eastern Finland, Kuopio, Finland; 7 Population Health Science, Bristol Medical School, University of Bristol and Medical Research Council Integrative Epidemiology Unit at the University of Bristol, Bristol, UK; 8 Systems Epidemiology, Baker Heart and Diabetes Institute, Melbourne, VIC Australia; 9 Department of Epidemiology and Preventive Medicine, School of Public Health and Preventive Medicine, Faculty of Medicine, Nursing and Health Sciences, The Alfred Hospital, Monash University, Melbourne, VIC Australia; 10 Inserm, UMRS 1138, Centre de Recherche des Cordeliers, Paris, France; 11 Assistance Publique Hôpitaux de Paris, Bichat Hospital, DHU FIRE, Department of Diabetology, Endocrinology and Nutrition, Paris, France; 12 University Paris Diderot, Sorbonne Paris Cité, UFR de Médecine, Paris, France; 13 International Centre for Circulatory Health, Imperial College, London, UK; 14 Department of Experimental Medicine, McGill University, Montreal, QC Canada; 15 Department of Medicine, CRCHUM, Université de Montréal, Montreal, QC Canada; 16 Department of Medicine, Gene Medicine Services, CRCHUM, Université de Montréal, Montreal, QC Canada; 17 The George Institute for Global Health, University of Oxford, Oxford, UK; 18 Department of Epidemiology, Johns Hopkins University, Baltimore, MD USA

**Keywords:** Amino acid, Diabetes complications, Metabolomics, Risk factors, Type 2 diabetes

## Abstract

**Aims/hypotheses:**

We aimed to quantify the association of individual circulating amino acids with macrovascular disease, microvascular disease and all-cause mortality in individuals with type 2 diabetes.

**Methods:**

We performed a case-cohort study (*N* = 3587), including 655 macrovascular events, 342 microvascular events (new or worsening nephropathy or retinopathy) and 632 all-cause mortality events during follow-up, in a secondary analysis of the Action in Diabetes and Vascular Disease: Preterax and Diamicron Modified Release Controlled Evaluation (ADVANCE) study. For this study, phenylalanine, isoleucine, glutamine, leucine, alanine, tyrosine, histidine and valine were measured in stored plasma samples by proton NMR metabolomics. Hazard ratios were modelled per SD increase in each amino acid.

**Results:**

In models investigating associations and potential mechanisms, after adjusting for age, sex and randomised treatment, phenylalanine was positively, and histidine inversely, associated with macrovascular disease risk. These associations were attenuated to the null on further adjustment for extended classical risk factors (including eGFR and urinary albumin/creatinine ratio). After adjustment for extended classical risk factors, higher tyrosine and alanine levels were associated with decreased risk of microvascular disease (HR 0.78; 95% CI 0.67, 0.91 and HR 0.86; 95% CI 0.76, 0.98, respectively). Higher leucine (HR 0.79; 95% CI 0.69, 0.90), histidine (HR 0.89; 95% CI 0.81, 0.99) and valine (HR 0.79; 95% CI 0.70, 0.88) levels were associated with lower risk of mortality. Investigating the predictive ability of amino acids, addition of all amino acids to a risk score modestly improved classification of participants for macrovascular (continuous net reclassification index [NRI] +35.5%, *p* < 0.001) and microvascular events (continuous NRI +14.4%, *p* = 0.012).

**Conclusions/interpretation:**

We report distinct associations between circulating amino acids and risk of different major complications of diabetes. Low tyrosine appears to be a marker of microvascular risk in individuals with type 2 diabetes independently of fundamental markers of kidney function.

**Electronic supplementary material:**

The online version of this article (10.1007/s00125-018-4619-x) contains peer-reviewed but unedited supplementary material, which is available to authorised users.

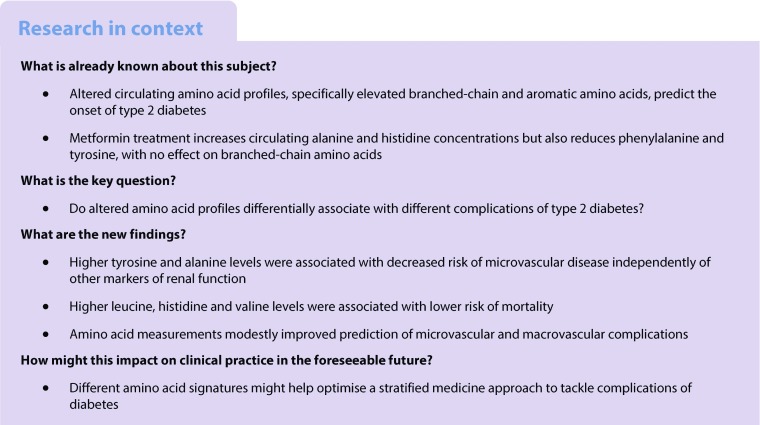



## Introduction

Prior to an individual developing overt type 2 diabetes, there appears to be a period of subclinical metabolic abnormality, manifesting in the altered circulating levels of many metabolites [1, 2]. Specifically, several studies have now reported that circulating concentrations of amino acids predict the development of type 2 diabetes. A nested case–control study from the Framingham Offspring study showed that branched-chain amino acids (BCAAs) isoleucine, leucine and valine and aromatic amino acids (AAAs) tyrosine and phenylalanine showed positive associations with insulin resistance and risk of type 2 diabetes [3]. The European Investigation into Cancer and Nutrition (EPIC) Potsdam study, the Metabolic Syndrome in Men (METSIM) study, the Cardiovascular Risk in Young Finns (CRY) study and the Southall and Brent Revisited (SABRE) study reported similar findings [4–7]. Glycine and glutamine have also been reported to be consistently inversely associated with risk of type 2 diabetes in a meta-analysis [8].

In general population studies, elevated levels of BCAAs and AAAs also appear to be associated with increased risk of cardiovascular disease [9–12], although these associations have not been entirely consistent [13]. In the large Estonian Biobank study, inverse associations between the concentration of several amino acids (including BCAAs) and all-cause mortality were observed [14]. An inverse association between BCAAs and clinical dementia or Alzheimer’s disease has also been observed [15]. Finally, we have recently reported in a randomised placebo-controlled trial that metformin treatment (for 18 months in men with CHD but without type 2 diabetes) led to improved insulin sensitivity and was associated with increases in alanine and histidine and reductions in phenylalanine and tyrosine concentrations, with no effect on BCAAs [16].

Therefore, the existing literature highlights inconsistent associations of amino acids with different outcomes in different studies, apparently contrary to the observations made in general population studies wherein elevated levels of BCAAs and AAAs are an adverse signal. This raises the possibility that the mechanisms that influence circulating amino acids might be more subtle than previously thought and as such it is worth investigating and contrasting the associations of measurable circulating amino acids with different adverse outcomes in people with type 2 diabetes.

A very small (*N* = 80) nested case–control study did not find that amino acids were associated with diabetic retinopathy [17]. However, we are aware of no large studies investigating the association of circulating amino acids with outcomes in individuals with type 2 diabetes.

Developing an understanding of any relationship between amino acids and a range of adverse outcomes in diabetes is important from an aetiological perspective, to develop hypotheses for intervention studies and potentially to develop clinical risk scores. We thus aimed to simultaneously investigate the association of circulating amino acids with the following outcomes in people with type 2 diabetes: (1) macrovascular disease; (2) microvascular disease and (3) all-cause mortality.

## Methods

### Participants

The Action in Diabetes and Vascular Disease: Preterax and Diamicron Modified Release Controlled Evaluation (ADVANCE) study (ClinicalTrials.gov registration no. NCT00145925) recruited 11,140 participants with type 2 diabetes between June 2001 and March 2003 [18]. Primary outcomes of the trial have been published [19, 20]. Participants were ≥55 years of age and had been diagnosed with type 2 diabetes after the age of 30 years. In addition, they were required to have a history of cardiovascular disease (CVD) or one or more additional cardiovascular risk factors. The trial included two randomised interventions: (1) a double-blind assessment of the efficacy of perindopril/indapamide (2 mg/0.625 mg for 3 months, increasing to 4 mg/1.25 mg if tolerated) vs placebo and (2) an open-label evaluation of an intensive glucose-lowering regimen using modified-release gliclazide (with a target HbA_1c_ of ≤48 mmol/mol [6.5%]) vs standard care. Participants had their serum creatinine levels measured as part of the study protocol at baseline, 4 months and 1 year and annually thereafter until completion of the study, with further tests at the discretion of clinicians. Urinary albumin/creatinine ratio (ACR) was measured as part of the study protocol at baseline, 2 years, 4 years and completion of the study. GFR was estimated using the Modification of Diet in Renal Disease formula. Participants underwent formal eye examination and visual acuity testing at baseline, 2 years, 4 years and completion of the study. Each participating centre obtained ethical approval, and all participants provided written informed consent.

The primary trial outcomes were composites of major macrovascular and microvascular events that occurred during a median of 5 years of follow-up. An independent adjudication committee validated all outcomes. Major macrovascular events were cardiovascular death, non-fatal myocardial infarction or non-fatal stroke. Major microvascular events were defined as a composite of new or worsening nephropathy or retinopathy, in turn defined as any of the following:

1. development of macroalbuminuria (urinary ACR >33.9 mg/mmol, confirmed by two results);
2. doubling of serum creatinine level to ≥200 μmol/l (with non-qualifying exceptions of terminal illness or acute illness and subsequent recovery of renal function);
3. the need for renal replacement therapy due to kidney disease (in the absence of other medical causes requiring transient dialysis), or death due to renal disease;
4. development of proliferative retinopathy (identified by the incidence of new blood vessels on the disc or elsewhere, vitreous haemorrhage, pre-retinal haemorrhage and fibrous proliferations on the disc or elsewhere in a participant found not to have this condition at entry);
5. development of macular oedema (characterised by a retinal thickening within one disc diameter of the macular centre in a participant not found to have this condition at entry);
6. occurrence of diabetes-related blindness (corrected visual acuity 3/60 or worse, persisting for ≥3 months and known to not be due to non-diabetes-related causes in a participant found not to have this condition at entry);
7. use of retinal photocoagulation therapy.

Blood samples were available from 17 out of 20 countries participating in the ADVANCE study (the exceptions were China, India and the Philippines), giving a total potential source cohort size for the study of 7376 individuals (66.2% of the overall study cohort).

**Fig. 1:**
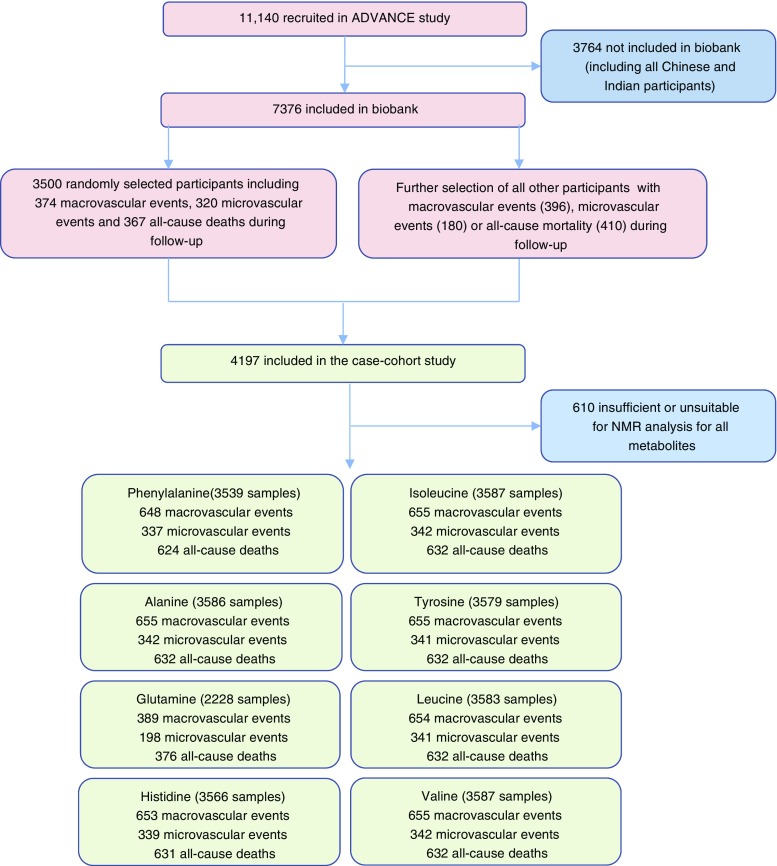
Flow diagram for design and sample analysis in the ADVANCE study of amino acids (note microvascular, macrovascular and all-cause mortality not mutually exclusive)

To improve efficiency of the biomarker studies in the ADVANCE trial a case-cohort study has been established [21, 22]. In case-cohort studies, a random sample (called the ‘subcohort’) is drawn and phenotyped from the full cohort; this is very likely to contain both individuals who are ‘cases’ and ‘non-cases’. Cases (generally for multiple case definitions, such as microvascular disease and macrovascular disease) who were not included in the subcohort are then identified from the remainder of the cohort and were also phenotyped. The case-cohort study has several advantages over the nested case–control design, including the ability to investigate multiple endpoints simultaneously. For this case-cohort study, a random subcohort (*n* = 3500) was selected from the base population, which was enriched by the addition of individuals who had a cardiovascular event, a microvascular event or died during follow-up, giving a total study size of 4197 (Fig. 1) [21, 22].

### Proton NMR analysis

Plasma samples were obtained at baseline from all study participants when they were in an unfasted state, given that these were people with type 2 diabetes at risk of hypoglycaemic episodes. Samples were collected across sites in a pragmatic fashion (commensurate with a multinational RCT) according to local facilities. Plasma samples were separated and stored centrally at −80°C until measurement. The present study used a previously unthawed aliquot of plasma for ^1^H-NMR analysis. ^1^H-NMR spectroscopy was performed on all available EDTA plasma samples from the ADVANCE case-cohort study at baseline using a low-volume (100 μl) variation of the quantitative ^1^H-NMR method (Nightingale Health, Helsinki, Finland) described previously [23, 24] and reviewed [25]. Sample spectra were analysed on a Bruker AVANCE III HD spectrometer to quantify a targeted list of metabolites, lipids and lipoproteins, as described previously [25]. This list included eight amino acids (alanine, glutamine, histidine, isoleucine, leucine, valine, phenylalanine and tyrosine), which are detectable using the method, and are not in ‘congested’ regions of the NMR spectrum where multiple metabolites overlap. Metabolomic analyses of plasma samples tend to yield lower analyte concentrations than serum, both by NMR spectroscopy and other methods, although plasma demonstrates better stability and reproducibility [26]. Samples with a low glutamine/glutamate ratio were excluded from analyses of glutamine associations. Levels of all other amino acids were consistent with published data.

### Statistical analysis

Continuous data with approximately normal distributions (including all amino acids) are presented as mean ± SD; those with skewed distributions are presented as median (with interquartile range). Categorical data are presented as *n* (%). Pearson correlations were used to explore associations of the amino acids with each other. Associations of amino acids with classical risk factors were investigated across quarters of the distribution of each amino acid.

Cox regression models were fitted using the STSELPRE procedure for case-cohort analyses (StataCorp, College Station, TX, USA). Models estimated HRs for a 1 SD increase in each amino acid with each of the endpoints. Two models, with different potential confounding variables, were fitted for each amino acid/outcome combination: model 1 with age, sex, region and randomised treatment; model 2 with, additionally, a prior macrovascular complication of diabetes (myocardial infarction, stroke, hospital admission for a transient ischaemic attack or for unstable angina, coronary or peripheral revascularisation, or amputation secondary to peripheral vascular disease), duration of diabetes, current smoking, systolic blood pressure, BMI, urinary ACR, eGFR, HbA_1c_, plasma glucose, total cholesterol, HDL-cholesterol, triacylglycerols, aspirin or other antiplatelet agent, statin or other lipid-lowering agent, β-blocker, ACE inhibitor or angiotensin receptor blocker, metformin use, history of heart failure, participation in moderate and/or vigorous exercise for >15 min at least once weekly, and high-sensitivity C-reactive protein (CRP). A third adjustment model, attempting to include all amino acids in the same model, resulted in collinearity and estimates were thus not available. Non-linearity was tested by comparing the deviances of linear and categorical models and by the inclusion of polynomial components (quadratic and cubic terms). Other analyses were performed using SAS v9.2 (SAS Institute, Cary, NC, USA). All *p* values reported are two-sided, with the 5% threshold used to determine significance.

For the random subcohort, the ability of amino acids to discriminate between those who will and those who will not go on to suffer each of the three adverse outcomes were estimated, in the context of model 2, using *c* statistics for 5 year risk, accounting for censoring. In addition, the ability of amino acids to reclassify participants according to 5 year risk, using the continuous net reclassification index (NRI), was assessed by methods suitable for survival data, using bootstrapping [27].

Primary results came from use of all available data; sensitivity analyses using only participants with complete data were also performed.

## Results

### Baseline associations

A maximum of 3587 samples had available data for at least one amino acid (Fig. 1). Due to the design of the multicentre study, there was some variability in sample processing time, leading to some samples having low glutamine/glutamate ratios. As such, fewer samples had a result for glutamine [28]. The detected absolute concentrations of amino acids were generally comparable with data from other studies (see electronic supplementary material [ESM] Table 1).

In general, the amino acids showed a broad range of correlations with each other. Taking extreme examples, leucine and glutamine were not correlated (*r* = 0.03, *p* = 0.23) but BCAAs leucine, isoleucine and valine were highly intercorrelated (*r* ≥ 0.67, *p* < 0.001) (Table 1). The associations of amino acids with classical CVD risk factors are shown in ESM Tables 2–9. Phenylalanine was positively associated with older age, baseline CVD, higher CRP and higher baseline high-sensitivity troponin T (hsTnT) and amino-terminal pro B-type natriuretic peptide (NT-proBNP). In contrast, the other AAA, tyrosine, showed inverse associations with HbA_1c_ and ACR and a positive association with eGFR. Histidine, alanine and glutamine showed inconsistent associations with classical risk factors. The BCAAs leucine, isoleucine and valine were inversely associated with age, HDL-cholesterol and NT-proBNP but were positively associated with CVD, male sex, BMI, triacylglycerols and HbA_1c_.

**Table 1:** Pearson correlations (*r*) of the amino acids with each other

Amino acid	Phenylalanine	Isoleucine	Glutamine	Leucine	Alanine	Tyrosine	Histidine
Isoleucine	0.32	–					
Glutamine	−0.05	0.05	–				
Leucine	0.34	0.89	0.03	–			
Alanine	0.2	0.44	0.13	0.44	–		
Tyrosine	0.4	0.25	0.13	0.29	0.26	–	
Histidine	0.16	0.24	0.43	0.2	0.27	0.17	–
Valine	0.27	0.67	0.13	0.75	0.34	0.29	0.25

All correlations have *p* values of <0.001, except for phenylalanine vs glutamine (*p* = 0.02), isoleucine vs glutamine (*p* = 0.03) and glutamine vs leucine (*p* = 0.23)

### Macrovascular disease, microvascular disease and all-cause mortality

Baseline risk factors associated with all three endpoints included male sex, increased duration of diabetes, history of macrovascular disease, higher systolic blood pressure, lower HDL-cholesterol, higher HbA_1c_, higher ACR and higher hsTnT and NT-proBNP (Table 2).

**Table 2:** Baseline characteristics of the cohort classified by outcome status

Characteristic	Macrovascular disease			Microvascular disease			All-cause mortality		
	Yes	No	*p* value	Yes	No	*p* value	Yes	No	*p* value
*N*	655	2932		342	3245		632	2955	
Age, years	68.92 ± 6.52	66.36 ± 6.51	<0.001	65.85 ± 6.38	66.93 ± 6.60	0.004	69.94 ± 6.56	66.17 ± 6.40	<0.001
Male sex	451 (68.9)	1719 (58.6)	<0.001	227 (66.4)	1943 (59.9)	0.020	439 (69.5)	1731 (58.6)	<0.001
Region									
ANZ/SEA	155 (23.7)	714 (24.4)	0.375	123 (36.0)	746 (23.0)	<0.001	120 (19.0)	749 (25.3)	<0.001
Canada	33 (5.0)	185 (6.3)		28 (8.2)	190 (5.9)		34 (5.4)	184 (6.2)	
Continental Europe	262 (40.0)	1157 (39.5)		91 (26.6)	1328 (40.9)		264 (41.8)	1155 (39.1)	
Northern Europe	205 (31.3)	876 (29.9)		100 (29.2)	981 (30.2)		214 (33.9)	867 (29.3)	
Duration of diabetes, years	9.19 ± 7.10	7.61 ± 6.29	<0.001	9.74 ± 6.89	7.71 ± 6.40	<0.001	9.24 ± 7.60	7.62 ± 6.17	<0.001
Current smoker	94 (14.4)	439 (15.0)	0.686	48 (14.0)	485 (14.9)	0.652	103 (16.3)	430 (14.6)	0.263
History of macrovascular disease	323 (49.3)	929 (31.7)	<0.001	77 (22.5)	276 (8.5)	<0.001	283 (44.8)	969 (32.8)	<0.001
History of heart failure	56 (8.5)	110 (3.8)	<0.001	13 (3.8)	153 (4.7)	0.445	61 (9.7)	105 (3.6)	<0.001
Participation in moderate or vigorous activity	266 (40.6)	1470 (50.1)	<0.001	164 (48.0)	1572 (48.4)	0.863	262 (41.5)	1474 (49.9)	<0.001
Diastolic BP, mmHg	81.55 ± 11.41	81.63 ± 10.74	0.863	81.73 ± 11.30	81.60 ± 10.82	0.831	80.55 ± 11.70	81.84 ± 10.67	0.007
Total cholesterol, mmol/l	5.11 ± 1.18	5.15 ± 1.17	0.500	5.16 ± 1.08	5.14 ± 1.18	0.737	5.06 ± 1.10	5.16 ± 1.18	0.058
HDL-cholesterol, mmol/l	1.17 ± 0.31	1.24 ± 0.33	<0.001	1.18 ± 0.31	1.23 ± 0.33	0.005	1.18 ± 0.31	1.23 ± 0.33	<0.001
Triacylglycerol, mmol/l	1.63 (1.20, 2.30)	1.70 (1.20, 2.36)	0.436	1.80 (1.27, 2.60)	1.69 (1.20, 2.31)	0.01	1.61 (1.20, 2.30)	1.70 (1.20, 2.36)	0.4912
HbA_1c_, mmol/mol	59.5 ± 17.2	56.9 ± 14.8		61.3 ± 17.5	56.9 ± 15.4		59.3 ± 16.8	56.9 ± 15.2	
HbA_1c_, %	7.59 ± 1.60	7.36 ± 1.39	<0.001	7.76 ± 1.60	7.36 ± 1.41	<0.001	7.58 ± 1.58	7.36 ± 1.40	<0.001
Glucose, mmol/l	8.61 ± 2.85	8.43 ± 2.68	0.115	9.04 ± 3.37	8.40 ± 2.62	<0.001	8.53 ± 2.88	8.45 ± 2.67	0.453
Urinary ACR, mg/mmol	2.4 (1.0, 8.0)	1.5 (0.7, 4.0)	<0.001	5.6 (1.6, 14.2)	1.5 (0.7, 3.8)	<0.001	2.4 (0.9, 7.6)	1.5 (0.7, 4.0)	<0.001
eGFR, ml min^−1^ 1.73 m^−2^	67.68 ± 17.61	72.70 ± 16.37	<0.001	69.97 ± 18.74	71.98 ± 16.48	0.034	66.58 ± 17.57	72.89 ± 16.32	<0.001
CRP, nmol/l	19.33 (9.05, 42.38)	16.67 (8.10, 38.48)	0.012	15.71 (8.48, 32.95)	17.43 (8.29, 39.33)	0.249	19.90 (9.71, 45.81)	16.86 (8.10, 37.71)	<0.001
hsTnT, pg/ml	9 (4, 17)	5 (1.50, 10)	<0.001	7 (1.50, 13)	5 (1.50, 11)	<0.001	10 (4, 18)	5 (1.50, 9)	<0.001
NT-proBNP, pg/ml	198 (74, 479)	74 (30, 169)	<0.001	107 (38, 260)	87 (34, 212)	0.010	201 (80, 506)	74 (30, 171)	<0.001
Medication use									
Aspirin or other antiplatelet agent	387 (59.1)	1380 (47.1)	<0.001	171 (50.0)	1596 (49.2)	0.774	352 (55.7)	1415 (47.9)	<0.001
Statin or other lipid-lowering agent	283 (43.2)	1311 (44.7)	0.484	158 (46.2)	1436 (44.3)	0.490	260 (41.1)	1334 (45.1)	0.066
β blocker	211 (32.2)	881 (30.0)	0.276	96 (28.1)	996 (30.7)	0.316	196 (31.0)	896 (30.3)	0.731
ACE inhibitor or angiotensin receptor blocker	418 (63.8)	1671 (57.0)	0.0014	232 (67.8)	1857 (57.2)	<0.001	395 (62.5)	1694 (57.3)	0.017
Amino acid level, mmol/l									
Phenylalanine	0.063 ± 0.010	0.061 ± 0.009	<0.001	0.062 ± 0.009	0.062 ± 0.009	0.1327	0.063 ± 0.010	0.061 ± 0.009	<0.001
Isoleucine	0.063 ± 0.017	0.062 ± 0.017	0.7429	0.065 ± 0.017	0.062 ± 0.017	0.0059	0.061 ± 0.017	0.063 ± 0.017	0.0643
Glutamine	0.373 ± 0.112	0.380 ± 0.109	0.2634	0.369 ± 0.118	0.380 ± 0.109	0.1696	0.367 ± 0.112	0.381 ± 0.109	0.0247
Leucine	0.081 ± 0.019	0.082 ± 0.020	0.0679	0.084 ± 0.020	0.082 ± 0.020	0.0518	0.079 ± 0.020	0.082 ± 0.020	<0.001
Alanine	0.366 ± 0.064	0.371 ± 0.065	0.0591	0.370 ± 0.066	0.370 ± 0.064	0.9372	0.361 ± 0.063	0.372 ± 0.065	<0.001
Tyrosine	0.053 ± 0.012	0.053 ± 0.011	0.9293	0.050 ± 0.012	0.053 ± 0.011	<0.001	0.052 ± 0.012	0.053 ± 0.011	0.1873
Histidine	0.049 ± 0.010	0.050 ± 0.009	0.0032	0.050 ± 0.009	0.050 ± 0.010	0.4117	0.048 ± 0.010	0.050 ± 0.009	<0.001
Valine	0.172 ± 0.035	0.175 ± 0.035	0.1355	0.177 ± 0.037	0.174 ± 0.035	0.1729	0.167 ± 0.036	0.176 ± 0.035	<0.001

Values are mean ±SD, median (interquartile range) or *n* (%)

ANZ, Australia and New Zealand; SEA, south-east Asia

**Fig. 2:**
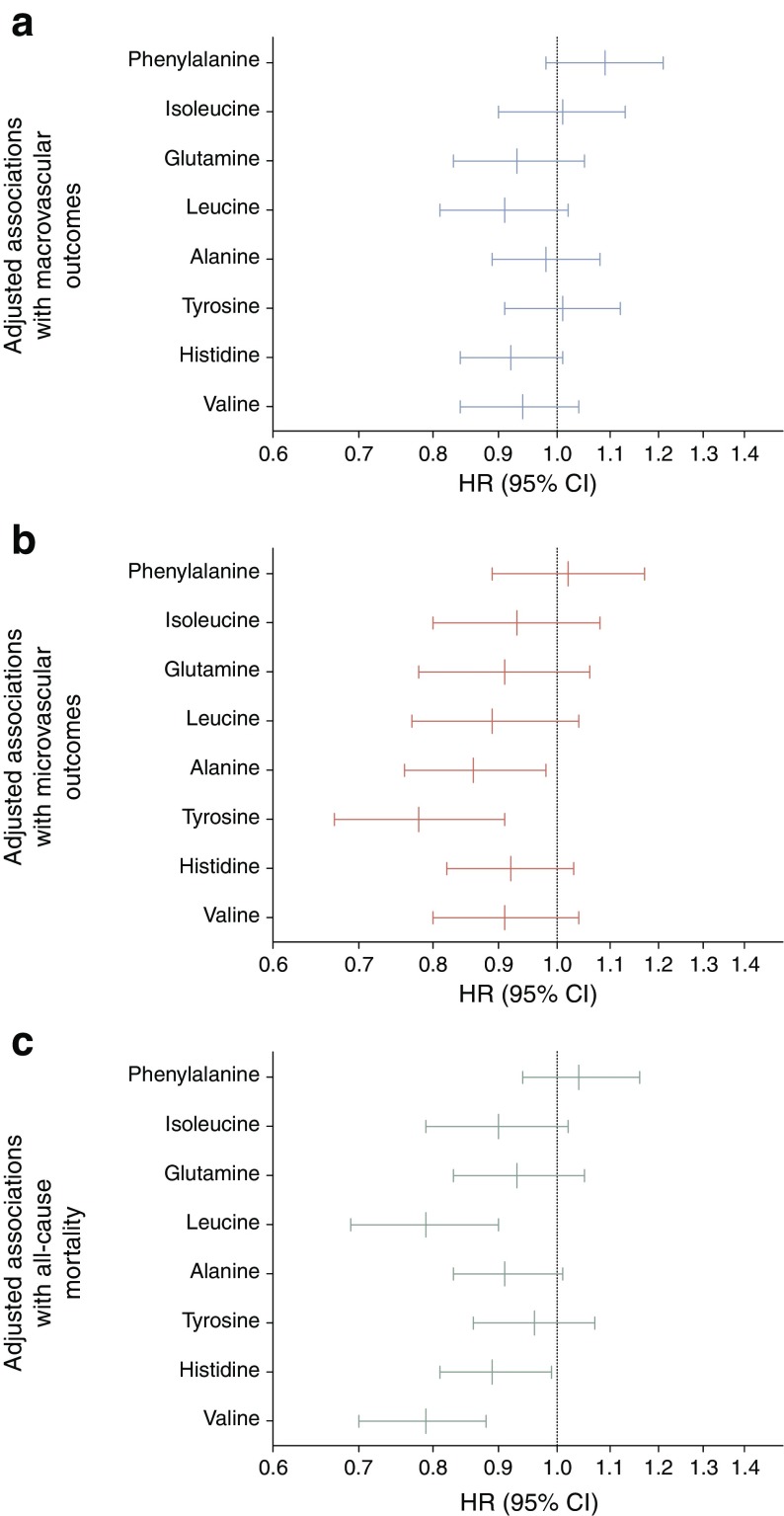
Adjusted associations (model 2, log_10_ scale HR) of amino acids individually (per 1 SD increase) with macrovascular outcomes (**a**), microvascular outcomes (**b**) and all-cause mortality (**c**)

Among the amino acids, after adjustment for age, sex, region and randomised treatment (model 1), higher phenylalanine and lower glutamine and histidine concentrations were associated with increased macrovascular risk (HR per 1 SD increase was 1.22 [95% CI 1.12, 1.32], 0.88 [95% CI 0.79, 0.98] and 0.86 [95% CI 0.79, 0.94], respectively) but these associations were attenuated to the null on further adjustment for classical risk factors (model 2) (Fig. 2a and ESM Table 10).

Higher tyrosine alone was associated with decreased risk of microvascular events (HR 0.74 [95% CI 0.64, 0.86]) in model 1 and this was only slightly attenuated on adjustment for a full range of classical risk factors in model 2 (HR 0.78 [95% CI 0.67, 0.91]) (Fig. 2b and ESM Table 10). A higher alanine level was also associated with decreased risk of microvascular events after further adjustment (HR 0.86 [95% CI 0.76, 0.98]). The association between tyrosine and renal impairment was further investigated by assessing the HRs across tertiles of eGFR and ACR. There was no evidence of interaction by eGFR or ACR (data not shown).

In contrast, several amino acids were associated with all-cause mortality. Phenylalanine was positively associated with risk of mortality, while glutamine, leucine, alanine, histidine and valine were all inversely associated with risk of mortality in model 1 (ESM Table 10). After adjustment for classical risk factors (model 2), the inverse association with risk remained for leucine (HR 0.79 [95% CI 0.69, 0.90]), histidine (HR 0.89 [95% CI 0.81, 0.99]) and valine (HR 0.79 [95% CI 0.70, 0.88]) but the positive association of phenylalanine was attenuated to the null (Fig. 2c and ESM Table 10). A sensitivity analysis using samples from individuals with complete data gave similar results (ESM Table 11). There was no evidence of a randomised treatment interaction in any model (data not shown).

A model including all the classical CVD risk factors in model 2 yielded a *c* statistic of 0.716 for macrovascular events, 0.728 for microvascular events and 0.747 for all-cause mortality (Table 3). Addition of the amino acids in combination did not improve the *c* statistic for any endpoint but did improve the continuous NRI for macrovascular events (+35.5%, *p* < 0.001) and microvascular events (+14.4%, *p* = 0.012). The improvement in prediction of microvascular events was driven by the addition of tyrosine alone.

**Table 3:** Prediction of endpoints using amino acids in combination or individually

Model	Macrovascular events		Microvascular events		All-cause mortality	
	*c* statistic	Continuous NRI (%)	*c* statistic	Continuous NRI (%)	*c* statistic	Continuous NRI (%)
Basic model^a^	0.716	–	0.728	–	0.747	–
Plus all amino acids	+0.010	+35.5	+0.010	+14.4	+0.009	+8.0
*p* value	0.17	<0.001	0.23	0.012	0.26	0.09
Plus tyrosine only	–	–	0.007	+14.9	–	–
*p* value	–	–	0.30	0.01	–	–

^a^Adjusted for age, sex, region, randomised treatment, previous macrovascular event, duration of diabetes, current smoking, systolic blood pressure, BMI, urinary ACR, eGFR, HbA_1c_, plasma glucose, total and HDL-cholesterol, triacylglycerols, use of aspirin or other antiplatelet agent, statin or other lipid-lowering agent, β-blocker, ACE inhibitor or angiotensin receptor blocker, or metformin, history of heart failure, participation in moderate and/or vigorous exercise for >15 min at least once weekly, and CRP

## Discussion

Although previous observational studies have reported associations of circulating BCAAs and AAAs with adverse outcomes in healthy people, the present report contrasts for the first time the associations of multiple circulating amino acids with the major vascular complications of diabetes. Rather than one (or more) amino acids being a consistent signal for adverse outcomes of any kind, we report that their associations with risk of macrovascular events, microvascular events and all-cause mortality are strikingly different from each other. A key finding is the inverse association of tyrosine with risk of microvascular events, independent of eGFR and urinary ACR. Although the evidence from the present study suggests that these might only be very moderately useful biomarkers in incremental prediction of adverse events in individuals with type 2 diabetes, the pathophysiology underlying these associations and the possibility of intervention studies are intriguing and worthy of further investigation.

The association of high circulating concentrations of BCAAs and AAAs with obesity has been known since the 1960s [29] and has been proposed to be at least partially mediated by insulin resistance. Insulin is thought to be a regulator of branched-chain α-keto acid dehydrogenase complex [30]. Insulin resistance may hence suppress BCAA catabolism, as suggested by associations noted in observational epidemiology studies [31–33]. The causal pathway may not be unidirectional; a recent Mendelian randomisation study suggests that genetically elevated BCAAs (via impaired catabolism) are associated with increased risk of type 2 diabetes [34], although a better understanding of the underlying pathway is required to increase confidence in this observation [35]. There is also the possibility that amino acids themselves (particularly BCAAs) may affect metabolism by suppressing postprandial glucose levels [36]. Increased protein turnover in people with central obesity may result in higher circulating levels of amino acids [37] and might therefore cause elevations in amino acids in people who are overweight and have type 2 diabetes. There are hence a variety of potential mechanisms related to type 2 diabetes pathologies that might influence circulating amino acids in individuals with type 2 diabetes.

Given this background, and prior findings in general populations of associations of specific amino acids with CVD [9–12], we wished to examine whether amino acid levels associated with adverse outcomes in individuals with type 2 diabetes. In the ADVANCE study, the BCAAs leucine, valine and isoleucine showed no association with macrovascular events, but low levels of leucine and valine were associated with increased all-cause mortality. However, the positive, albeit not independently predictive, association of phenylalanine with CVD and all-cause mortality we observed is broadly in line with other published data. There are limited intervention studies investigating the effect of amino acid supplements on health outcomes, with most research coming from short-term trials examining surrogate health markers in the sports science area [38]. Our data strongly support the need for further studies to determine why higher phenylalanine appears to be a consistently adverse signal for CVD outcomes. Our study provides observations that are the basis for testable hypotheses investigating the effect of genetic variants, which are instrumental variables for circulating amino acids, on health outcomes [39, 40].

The inverse association of tyrosine with risk of microvascular events is perhaps the most intriguing individual finding from this study. Tyrosine itself was positively associated with baseline eGFR and inversely associated with baseline HbA_1c_ and urinary ACR. Impaired conversion of phenylalanine to tyrosine has been reported in renal disease [41, 42]. Low tyrosine levels might therefore simply reflect impaired kidney function, which itself predicts future microvascular events. Tyrosine is also linked to catecholamine synthesis, which, also speculatively, might be relevant to our findings [43]. That noted, counter-intuitively, we have reported that metformin in fact lowers, not raises, tyrosine levels in individuals with CVD and at high risk of diabetes [16]. The effect of other glucose-lowering drugs on amino acid profiles in individuals with diabetes would now be of interest. Further studies are now needed to validate our novel observations and to examine whether our findings may represent causal pathways.

Strengths of the study include the use of a well-characterised clinical trial cohort, an efficiently designed case-cohort study to yield a powerful study for a range of endpoints, which were independently adjudicated according to pre-defined criteria. Like other RCT populations, ADVANCE study participants represent a selected cohort. For instance, ADVANCE study participants were required to have a history of CVD or CVD risk factors. Therefore, our results may not be generalisable to all individuals with diabetes, although other risk factors we measured are generally associated with risk of major endpoints in the expected directions. Amino acids were measured in pragmatically collected plasma samples in the context of a multinational RCT and we cannot rule out the potential for differential pre-analytical sample handling or sample degradation during storage, which may have biased our results [44], although these samples were analysed at first thaw. We also present data suggesting broadly comparable concentrations of amino acids relative to other cohorts. Another potential limitation is the analysis of samples from non-fasted participants, although in clinical practice, fasting is rarely required among individuals with type 2 diabetes. NMR spectroscopy has been used to investigate changes in amino acids 30 min after a standardised liquid meal [45] and effects sizes were generally relatively small, although the immediate postprandial state is likely to give larger effect estimates than are at play in this study.

In conclusion, we report distinct associations of different amino acids with risk of major adverse endpoints in individuals with type 2 diabetes. Most notably, the identification of tyrosine as a potential marker of microvascular risk requires further study.

## Electronic supplementary material

ESM Tables (PDF 659 kb)

## Data Availability

Summaries of the ADVANCE trial data can be found at http://www.advance-trial.com. Restrictions apply to the availability of these data, which were used by agreement of the ADVANCE steering committee for the current study, and so are not publicly available.

## Abbreviations

AAA: Aromatic amino acid
ACR: Albumin/creatinine ratio
ADVANCE: Action in Diabetes and Vascular Disease: Preterax and Diamicron Modified Release Controlled Evaluation
BCAA: Branched-chain amino acid
CRP: C-reactive protein
CVD: Cardiovascular disease
hsTnT: High-sensitivity troponin T
NRI: Net reclassification index
NT-proBNP: Amino-terminal pro B-type natriuretic peptide
