# Supplementary material for: Circulating amino acids and the risk of macrovascular, microvascular and mortality outcomes in individuals with type 2 diabetes: results from the ADVANCE trial
Source: Diabetologia. 2018 May 4;61(7):1581–91. doi: 10.1007/s00125-018-4619-x (PMC6445481; doi:10.1007/s00125-018-4619-x)
Supplement: Supplementary file 1 — (PDF 659 kb) [file 125_2018_4619_MOESM1_ESM.pdf]

Circulating amino acids and the risk of macrovascular, microvascular, and mortality outcomes in individuals with type 2 diabetes: results from the ADVANCE trial

Welsh et al

SUPPLEMENTARY DATA

ESM Table 1 Illustrative comparisons of mean or median concentrations of amino acids measured by comparable method (1), by study.

Study	**Phenylalanine (mmol/l)**	**Isoleucine (mmol/l)**	**Glutamine (mmol/l)**	**Leucine (mmol/l)**	**Alanine (mmol/l)**	**Tyrosine (mmol/l)**	**Histidine (mmol/l)**	**Valine (mmol/l)**
ADVANCE: participant with type 2 diabetes case-cohort study.<br>2955 who did not die during follow-up.<br>Mean (SD)	0.061<br>(0.009)	0.063<br>(0.017)	0.381<br>(0.109)	0.082<br>(0.020)	0.372<br>(0.065)	0.053<br>(0.011)	0.050<br>(0.009)	0.176<br>(0.035)
SABRE (2): A community-based cohort of European, South Asian and African-Caribbean origin in London.<br>1279 Europeans without diabetes.<br>Median (IQR)	0.092<br>(0.084,0.100)	0.057<br>(0.050,0.066)	0.384<br>(0.257,0.448)	0.091<br>(0.080,0.110)	0.326<br>(0.290,0.365)	0.054<br>(0.048,0.061)	0.077<br>(0.069,0.088)	0.179<br>(0.158,0.202)
SABRE (2): A community-based cohort of European, South Asian and African-Caribbean origin in London.<br>1007 South Asians without diabetes.<br>Median (IQR)	0.094<br>(0.086,0.100)	0.060<br>(0.052,0.068)	0.410<br>(0.309,0.471)	0.093<br>(0.083,0.110)	0.335<br>(0.301,0.375)	0.060<br>(0.053,0.067)	0.078<br>(0.070,0.089)	0.178<br>(0.156,0.199)
FINRISK (3): Finnish general population cohort.<br>7610 with available serum sample.<br>Mean (SD)	0.085<br>(0.014)	0.058<br>(0.019)	0.470<br>(0.070)	0.088<br>(0.021)	0.430<br>(0.067)	0.051<br>(0.013)	0.064<br>(0.010)	0.210<br>(0.043)
Young Finns Study (4): Cohort study of younger adults aged 34-49.<br>1630 with normal fatty liver score.<br>Mean (SD)	0.074<br>(0.010)	0.051<br>(0.015)	0.595<br>(0.068)	0.080<br>(0.016)	0.399<br>(0.059)	0.050<br>(0.010)	0.065<br>(0.010)	0.199<br>(0.038)
CAMERA Trial (5): Randomised controlled trial of metformin in people with CHD but not diabetes.<br>173 randomised participants<br>Mean (SD)	0.047<br>(0.008)	0.043<br>(0.013)	0.396<br>(0.064)	0.056<br>(0.012)	0.250<br>(0.062)	0.061<br>(0.012)	0.046<br>(0.010)	0.152<br>(0.031)

ESM Table 2 Baseline characteristics classified by quarters of the phenylalanine distribution

	Phenylalanine (mmol\L)				
**Characteristics**	**1st(<=0.056) (N = 885)**	**2nd(0.056 to <=0.061) (N = 886)**	**3rd(0.061 to <=0.066) (N = 884)**	**4th(>0.066) (N = 884)**	**p-value for trend**
Sex					
Male	545/ 885 (61.6%)	527/ 886 (59.5%)	525/ 884 (59.4%)	553/ 884 (62.6%)	0.6999
Female	340/ 885 (38.4%)	359/ 886 (40.5%)	359/ 884 (40.6%)	331/ 884 (37.4%)	
Current smokers					
No	747/ 885 (84.4%)	763/ 886 (86.1%)	745/ 884 (84.3%)	757/ 884 (85.6%)	0.7314
Yes	138/ 885 (15.6%)	123/ 886 (13.9%)	139/ 884 (15.7%)	127/ 884 (14.4%)	
History of macrovascular event					
No	626/ 885 (70.7%)	580/ 886 (65.5%)	538/ 884 (60.9%)	556/ 884 (62.9%)	<0.001
Yes	259/ 885 (29.3%)	306/ 886 (34.5%)	346/ 884 (39.1%)	328/ 884 (37.1%)	
Age (years)					
N	885	886	884	884	
Mean (SD)	66.0 (6.4)	66.4 (6.4)	66.9 (6.5)	68.0 (6.8)	<0.001
Median (IRQ)	66.0 (61.0, 71.0)	67.0 (61.0, 71.0)	67.0 (62.0, 71.0)	68.0 (63.0, 73.0)	
Duration of diabetes (years)					
N	885	885	884	884	
Mean (SD)	8.5 (6.6)	7.8 (6.3)	7.8 (6.3)	7.5 (6.6)	0.001
Median (IRQ)	7.0 (3.0, 12.0)	6.0 (3.0, 11.0)	6.0 (3.0, 11.0)	6.0 (2.0, 11.0)	
BMI (Kg/m2)					
N	885	885	883	884	
Mean (SD)	28.8 (4.5)	29.9 (5.1)	30.6 (5.6)	30.8 (5.5)	<0.001

Characteristics	Phenylalanine (mmol\L)<br>1st(<=0.056)<br>(N = 885)	2nd(0.056 to <=0.061)<br>(N = 886)	3rd(0.061 to <=0.066)<br>(N = 884)	4th(>0.066)<br>(N = 884)	p-value<br>for trend
Median (IRQ)	28.4 (25.7, 31.3)	29.1 (26.5, 32.5)	29.7 (26.7, 33.5)	30.1 (27.0, 33.6)	
History of heart failure					
No	859/ 885 (97.1%)	855/ 886 (96.5%)	839/ 884 (94.9%)	824/ 884 (93.2%)	<0.001
Yes	26/ 885 (2.9%)	31/ 886 (3.5%)	45/ 884 (5.1%)	60/ 884 (6.8%)	
Participation in moderate or vigorous activity					
No	439/ 885 (49.6%)	431/ 886 (48.6%)	453/ 884 (51.2%)	499/ 884 (56.4%)	0.0021
Yes	446/ 885 (50.4%)	455/ 886 (51.4%)	431/ 884 (48.8%)	385/ 884 (43.6%)	
SBP (mmHg)					
N	885	886	883	884	
Mean (SD)	146.1 (21.1)	147.6 (21.9)	148.2 (21.8)	146.7 (21.0)	0.4541
Median (IRQ)	144.0 (131.5, 158.0)	145.0 (132.5, 159.5)	146.5 (133.5, 160.5)	145.5 (132.5, 159.0)	
DBP (mmHg)					
N	885	886	883	884	
Mean (SD)	81.3 (10.9)	81.9 (10.7)	82.4 (11.0)	80.8 (10.7)	0.5481
Median (IRQ)	80.5 (73.5, 88.5)	81.5 (74.5, 89.0)	82.0 (75.0, 89.0)	80.5 (73.5, 87.5)	
Total chol (mmol/l)					
N	885	884	884	884	
Mean (SD)	5.1 (1.1)	5.2 (1.2)	5.2 (1.3)	5.1 (1.1)	0.1233
Median (IRQ)	5.0 (4.3, 5.9)	5.1 (4.3, 5.9)	5.0 (4.4, 5.9)	4.9 (4.3, 5.8)	
HDL Chol (mmol/l)					
N	884	883	884	883	

Characteristics	Phenylalanine (mmol\L) 1st(<=0.056) (N = 885)	2nd(0.056 to <=0.061) (N = 886)	3rd(0.061 to <=0.066) (N = 884)	4th(>0.066) (N = 884)	p-value for trend
Mean (SD)	1.3 (0.3)	1.2 (0.3)	1.2 (0.3)	1.2 (0.3)	<0.001
Median (IRQ)	1.2 (1.0, 1.5)	1.2 (1.0, 1.4)	1.2 (1.0, 1.4)	1.2 (1.0, 1.4)	
Triacylglycerol (mmol/l)					
N	885	884	882	883	
Mean (SD)	1.8 (1.1)	2.0 (1.3)	2.0 (1.1)	2.0 (1.3)	0.0017
Median (IRQ)	1.6 (1.2, 2.2)	1.7 (1.2, 2.3)	1.8 (1.3, 2.4)	1.7 (1.3, 2.4)	
HbA1c					
N	882	881	881	879	
Mean % (SD) [mmol/mol]	7.3 (1.3) [52]	7.4 (1.4) [57]	7.5 (1.5) [59]	7.4 (1.5) [57]	0.1989
Urinary albumin:creatinine ratio (mg/mmol)					
N	823	838	845	819	
Median (IRQ)	1.4 (0.7, 3.6)	2 (0.8, 5.3)	2 (0.8, 5.3)	2.2 (0.9, 6.5)	<0.001
Glucose (mmol/l)					
N	885	884	884	884	
Mean (SD)	8.5 (2.6)	8.5 (2.6)	8.6 (2.8)	8.3 (2.8)	0.1697
Median (IRQ)	8.1 (6.8, 9.7)	8.0 (6.6, 9.8)	8.0 (6.6, 9.9)	7.7 (6.4, 9.6)	
eGFR (mL/min/1.73 m²)					
N	883	883	878	878	
Mean (SD)	75.4 (14.9)	73.2 (16.5)	70.8 (17.1)	68.0 (17.4)	<0.001
Median (IRQ)	75.7 (64.9, 87.5)	73.8 (60.6, 87.3)	70.8 (59.2, 83.9)	67.4 (56.7, 81.4)	

	Phenylalanine (mmol\L)				
**Characteristics**	**1st(<=0.056) (N = 885)**	**2nd(0.056 to <=0.061) (N = 886)**	**3rd(0.061 to <=0.066) (N = 884)**	**4th(>0.066) (N = 884)**	**p-value for trend**
CRP (nmol/l)					
N	871	856	861	867	
Mean (SD)	21.9 (31.4)	29.5 (49.5)	36.2 (70.5)	51.4 (93.3)	<0.001
Median (IRQ)	12.4 (6.7, 24.8)	15.2 (8.6, 37.1)	20.0 (9.5, 41.9)	24.8 (10.5, 51.4)	
hs-cTnT (ng/l)					
N	870	856	860	866	
Mean (SD)	7.8 (12.9)	7.5 (8.7)	8.9 (17.8)	11.4 (32.0)	<0.001
Median (IRQ)	4.0 (1.5, 9.0)	5.0 (1.5, 10.0)	6.0 (1.5, 11.0)	6.0 (1.5, 13.0)	
NT-proBNP (pg/mL)					
N	870	856	860	866	
Mean (SD)	177.4 (556.8)	206.3 (418.9)	259.8 (557.4)	366.4 (1487.4)	<0.001
Median (IRQ)	67.0 (29.0, 159.0)	83.0 (31.0, 202.5)	97.0 (39.5, 247.5)	113.0 (40.0, 281.0)	

Values are n (%), mean (standard deviation), or median (interquartile range)

ESM Table 3 Baseline characteristics classified by quarters of the isoleucine distribution

	Isoleucine (mmol\L)				
**Characteristics**	**1st(<=0.051) (N = 897)**	**2nd(0.051 to <=0.060) (N = 900)**	**3rd(0.060 to <=0.071) (N = 894)**	**4th(>0.071) (N = 896)**	**p-value for trend**
Sex					
Male	426/ 897 (47.5%)	522/ 900 (58.0%)	578/ 894 (64.7%)	644/ 896 (71.9%)	<0.001
Female	471/ 897 (52.5%)	378/ 900 (42.0%)	316/ 894 (35.3%)	252/ 896 (28.1%)	
Current smokers					
No	788/ 897 (87.8%)	778/ 900 (86.4%)	738/ 894 (82.6%)	750/ 896 (83.7%)	0.0022
Yes	109/ 897 (12.2%)	122/ 900 (13.6%)	156/ 894 (17.4%)	146/ 896 (16.3%)	
History of macrovascular event					
No	640/ 897 (71.3%)	592/ 900 (65.8%)	554/ 894 (62.0%)	549/ 896 (61.3%)	<0.001
Yes	257/ 897 (28.7%)	308/ 900 (34.2%)	340/ 894 (38.0%)	347/ 896 (38.7%)	
Age (years)					
N	897	900	894	896	
Mean (SD)	67.9 (6.8)	67.6 (6.3)	66.7 (6.4)	65.2 (6.5)	<0.001
Median (IRQ)	68.0 (63.0, 72.0)	68.0 (63.0, 72.0)	67.0 (61.0, 71.0)	65.0 (60.0, 70.0)	
Duration of diabetes (years)					
N	897	900	893	896	
Mean (SD)	8.1 (7.0)	7.8 (6.3)	7.8 (6.4)	8.0 (6.1)	0.7730
Median (IRQ)	6.0 (3.0, 11.0)	6.0 (3.0, 11.0)	6.0 (3.0, 11.0)	6.5 (3.0, 12.0)	
BMI (Kg/m2)					
N	897	900	892	896	
Mean (SD)	28.8 (5.3)	29.9 (5.2)	30.5 (5.4)	30.8 (4.9)	<0.001

	Isoleucine (mmol\L)				
Characteristics	1st(<=0.051)<br>(N = 897)	2nd(0.051 to <=0.060)<br>(N = 900)	3rd(0.060 to <=0.071)<br>(N = 894)	4th(>0.071)<br>(N = 896)	p-value<br>for trend
Median (IRQ)	28.0 (25.2, 31.6)	29.3 (26.5, 32.9)	29.8 (27.0, 33.2)	30.1 (27.4, 33.3)	
History of heart failure					
No	858/ 897 (95.7%)	850/ 900 (94.4%)	858/ 894 (96.0%)	855/ 896 (95.4%)	0.7875
Yes	39/ 897 (4.3%)	50/ 900 (5.6%)	36/ 894 (4.0%)	41/ 896 (4.6%)	
Participation in moderate or vigorous activity					
No	473/ 897 (52.7%)	440/ 900 (48.9%)	474/ 894 (53.0%)	464/ 896 (51.8%)	0.8619
Yes	424/ 897 (47.3%)	460/ 900 (51.1%)	420/ 894 (47.0%)	432/ 896 (48.2%)	
SBP (mmHg)					
N	897	900	893	896	
Mean (SD)	146.0 (22.1)	147.3 (21.8)	148.0 (21.5)	147.4 (20.6)	0.1452
Median (IRQ)	144.0 (130.5, 158.0)	145.0 (133.0, 159.5)	145.0 (133.5, 160.0)	146.5 (133.0, 160.0)	
DBP (mmHg)					
N	897	900	893	896	
Mean (SD)	80.0 (10.7)	81.6 (10.7)	82.3 (11.1)	82.5 (10.8)	<0.001
Median (IRQ)	80.0 (72.5, 87.0)	81.0 (74.5, 88.0)	81.5 (75.0, 89.0)	82.0 (75.0, 90.0)	
Total chol (mmol/l)					
N	897	900	893	895	
Mean (SD)	5.1 (1.1)	5.1 (1.1)	5.1 (1.1)	5.3 (1.3)	0.0092
Median (IRQ)	5.0 (4.3, 5.8)	5.0 (4.3, 5.8)	5.0 (4.3, 5.8)	5.2 (4.4, 6.0)	
HDL Chol (mmol/l)					
N	895	900	893	894	

	Isoleucine (mmol\L)				
**Characteristics**	**1st(<=0.051) (N = 897)**	**2nd(0.051 to <=0.060) (N = 900)**	**3rd(0.060 to <=0.071) (N = 894)**	**4th(>0.071) (N = 896)**	**p-value for trend**
Mean (SD)	1.3 (0.4)	1.3 (0.3)	1.2 (0.3)	1.1 (0.3)	<0.001
Median (IRQ)	1.3 (1.1, 1.6)	1.2 (1.0, 1.4)	1.2 (1.0, 1.4)	1.1 (0.9, 1.3)	
Triacylglycerol (mmol/l)					
N	897	900	893	892	
Mean (SD)	1.4 (0.6)	1.7 (0.7)	2.0 (1.0)	2.8 (1.7)	<0.001
Median (IRQ)	1.2 (0.9, 1.6)	1.5 (1.2, 2.0)	1.8 (1.4, 2.4)	2.5 (1.8, 3.4)	
HbA1c (%)					
N	894	896	889	892	
Mean % (SD) [mmol/mol]	7.2 (1.3) [55]	7.3 (1.4) [56]	7.5 (1.4) [59]	7.7 (1.5) [61]	<0.001
Urinary albumin:creatinine ratio (mg/mmol)					
N	842	853	844	833	
Median (IRQ)	1.7 (0.8, 4.5)	1.8 (0.8, 4.9)	2.1 (0.9, 5.4)	2.1 (0.9, 6.5)	0.2396
Glucose (mmol/l)					
N	897	900	893	895	
Mean (SD)	7.9 (2.5)	8.1 (2.5)	8.6 (2.7)	9.2 (3.0)	<0.001
Median (IRQ)	7.5 (6.4, 8.9)	7.7 (6.4, 9.2)	8.0 (6.7, 9.8)	8.8 (7.0, 10.7)	
eGFR (mL/min/1.73 m²)					
N	894	898	889	889	
Mean (SD)	70.8 (16.4)	71.7 (16.8)	72.8 (16.3)	71.8 (17.2)	0.0930
Median (IRQ)	71.2 (59.2, 83.8)	72.3 (60.6, 85.5)	72.9 (61.8, 85.0)	71.5 (59.7, 85.7)	

Characteristics	Isoleucine (mmol\L)<br>1st(<=0.051)<br>(N = 897)	2nd(0.051 to <=0.060)<br>(N = 900)	3rd(0.060 to <=0.071)<br>(N = 894)	4th(>0.071)<br>(N = 896)	p-value<br>for trend
CRP (nmol/l)					
N	874	877	866	874	
Mean (SD)	41.9 (97.1)	30.5 (57.1)	36.2 (59.0)	31.4 (41.9)	0.0091
Median (IRQ)	15.2 (7.6, 40.0)	17.1 (8.6, 37.1)	18.1 (8.6, 41.0)	19.0 (9.5, 39.0)	
hs-cTnT (ng/l)					
N	873	877	864	874	
Mean (SD)	8.4 (12.2)	8.2 (10.2)	9.9 (31.8)	9.1 (18.0)	0.2203
Median (IRQ)	5.0 (1.5, 11.0)	5.0 (1.5, 11.0)	5.0 (1.5, 11.0)	6.0 (1.5, 11.0)	
NT-proBNP (pg/mL)					
N	873	877	864	874	
Mean (SD)	324.9 (1392.9)	268.0 (609.5)	223.3 (469.3)	197.6 (703.4)	0.0012
Median (IRQ)	103.0 (46.0, 252.0)	89.0 (35.0, 222.0)	85.0 (30.5, 212.0)	70.0 (27.0, 179.0)	

Values are n (%), mean (standard deviation), or median (interquartile range)

ESM Table 4 Baseline characteristics classified by quarters of the glutamine distribution

	Glutamine (mmol\L)				
**Characteristics**	**1st(<=0.310) (N = 557)**	**2nd(0.310 to <=0.396) (N = 557)**	**3rd(0.396 to <=0.456) (N = 557)**	**4th(>0.456) (N = 557)**	**p-value for trend**
Sex					
Male	307/ 557 (55.1%)	347/ 557 (62.3%)	363/ 557 (65.2%)	365/ 557 (65.5%)	<0.001
Female	250/ 557 (44.9%)	210/ 557 (37.7%)	194/ 557 (34.8%)	192/ 557 (34.5%)	
Current smokers					
No	482/ 557 (86.5%)	467/ 557 (83.8%)	473/ 557 (84.9%)	471/ 557 (84.6%)	0.4742
Yes	75/ 557 (13.5%)	90/ 557 (16.2%)	84/ 557 (15.1%)	86/ 557 (15.4%)	
History of macrovascular event					
No	378/ 557 (67.9%)	364/ 557 (65.4%)	347/ 557 (62.3%)	338/ 557 (60.7%)	0.0069
Yes	179/ 557 (32.1%)	193/ 557 (34.6%)	210/ 557 (37.7%)	219/ 557 (39.3%)	
Age (years)					
N	557	557	557	557	
Mean (SD)	66.7 (6.6)	66.5 (6.5)	66.7 (6.4)	67.0 (6.6)	0.3772
Median (IRQ)	67.0 (62.0, 72.0)	67.0 (61.0, 71.0)	67.0 (62.0, 71.0)	67.0 (62.0, 72.0)	
Duration of diabetes (years)					
N	557	557	557	557	
Mean (SD)	8.5 (6.8)	8.0 (6.5)	7.1 (5.9)	7.4 (6.3)	<0.001
Median (IRQ)	7.0 (3.0, 12.0)	7.0 (3.0, 11.0)	6.0 (2.0, 10.0)	6.0 (2.0, 11.0)	
BMI (Kg/m2)					
N	557	555	557	557	
Mean (SD)	30.1 (4.9)	30.5 (5.5)	29.9 (5.2)	28.8 (5.1)	<0.001

Characteristics	Glutamine (mmol\L)<br>1st(<=0.310)<br>(N = 557)	2nd(0.310 to <=0.396)<br>(N = 557)	3rd(0.396 to <=0.456)<br>(N = 557)	4th(>0.456)<br>(N = 557)	p-value for trend
Median (IRQ)	29.7 (26.7, 32.9)	29.7 (26.6, 33.5)	29.2 (26.5, 32.5)	27.9 (25.5, 31.1)	
History of heart failure					
No	529/ 557 (95.0%)	536/ 557 (96.2%)	539/ 557 (96.8%)	544/ 557 (97.7%)	0.0154
Yes	28/ 557 (5.0%)	21/ 557 (3.8%)	18/ 557 (3.2%)	13/ 557 (2.3%)	
Participation in moderate or vigorous activity					
No	273/ 557 (49.0%)	310/ 557 (55.7%)	278/ 557 (49.9%)	284/ 557 (51.0%)	0.9849
Yes	284/ 557 (51.0%)	247/ 557 (44.3%)	279/ 557 (50.1%)	273/ 557 (49.0%)	
SBP (mmHg)					
N	557	557	557	557	
Mean (SD)	146.7 (21.6)	147.1 (20.9)	145.9 (21.3)	145.5 (22.3)	0.2289
Median (IRQ)	145.5 (131.0, 160.0)	145.5 (133.5, 158.5)	143.5 (131.5, 158.0)	143.5 (130.0, 159.0)	
DBP (mmHg)					
N	557	557	557	557	
Mean (SD)	81.3 (10.8)	81.4 (10.8)	81.1 (10.3)	80.5 (10.8)	0.1532
Median (IRQ)	81.0 (74.0, 88.5)	81.5 (74.0, 88.5)	81.0 (74.0, 88.0)	80.0 (73.0, 87.5)	
Total chol (mmol/l)					
N	556	557	556	557	
Mean (SD)	5.1 (1.1)	5.1 (1.0)	5.0 (1.1)	5.0 (1.1)	0.0449
Median (IRQ)	5.1 (4.3, 5.9)	5.0 (4.4, 5.8)	4.9 (4.3, 5.7)	4.9 (4.3, 5.7)	
HDL Chol (mmol/l)					
N	556	557	555	557	

Characteristics	Glutamine (mmol\L) 1st(<=0.310) (N = 557)	2nd(0.310 to <=0.396) (N = 557)	3rd(0.396 to <=0.456) (N = 557)	4th(>0.456) (N = 557)	p-value for trend
Mean (SD)	1.2 (0.3)	1.2 (0.3)	1.2 (0.3)	1.3 (0.4)	0.0011
Median (IRQ)	1.1 (1.0, 1.4)	1.2 (1.0, 1.4)	1.2 (1.0, 1.4)	1.2 (1.0, 1.5)	
Triacylglycerol (mmol/l)					
N	555	557	556	557	
Mean (SD)	2.0 (1.3)	2.0 (1.3)	1.9 (0.9)	1.8 (1.0)	<0.001
Median (IRQ)	1.7 (1.2, 2.4)	1.7 (1.3, 2.4)	1.7 (1.3, 2.3)	1.5 (1.1, 2.1)	
HbA1c (%)					
N	556	556	555	555	
Mean % (SD) [mmol/mol]	7.6 (1.6) [60]	7.4 (1.4) [57]	7.3 (1.2) [56]	6.9 (1.0) [52]	<0.001
Urinary albumin:creatinine ratio (mg/mmol)					
N	522	520	527	529	
Median (IRQ)	1.9 (0.9, 5.5)	1.6 (0.7, 5.8)	1.5 (0.7, 4)	1.5 (0.7, 4.4)	0.0977
Glucose (mmol/l)					
N	556	556	557	557	
Mean (SD)	8.9 (3.1)	8.6 (2.8)	8.4 (2.4)	7.6 (1.9)	<0.001
Median (IRQ)	8.2 (6.8, 10.3)	8.1 (6.7, 10.2)	8.0 (6.7, 9.7)	7.4 (6.3, 8.5)	
eGFR (mL/min/1.73 m^2)					
N	554	556	554	554	
Mean (SD)	70.9 (16.6)	71.7 (16.9)	72.0 (15.5)	71.7 (16.6)	0.3863
Median (IRQ)	69.9 (59.4, 83.6)	70.1 (59.6, 86.1)	72.2 (60.9, 84.3)	72.5 (61.4, 83.9)	

	Glutamine (mmol\L)				
Characteristics	1st(<=0.310)<br>(N = 557)	2nd(0.310 to <=0.396)<br>(N = 557)	3rd(0.396 to <=0.456)<br>(N = 557)	4th(>0.456)<br>(N = 557)	p-value<br>for trend
CRP (nmol/l)					
N	533	544	545	543	
Mean (SD)	41.9 (97.1)	42.9 (68.6)	28.6 (37.1)	21.9 (30.5)	<0.001
Median (IRQ)	19.0 (8.6, 42.9)	21.0 (9.5, 45.7)	17.1 (9.5, 36.2)	12.4 (6.7, 24.8)	
hs-cTnT (ng/l)					
N	533	544	544	542	
Mean (SD)	11.2 (43.0)	8.9 (14.7)	7.9 (9.5)	8.5 (10.3)	0.0433
Median (IRQ)	5.0 (1.5, 11.0)	5.0 (1.5, 11.0)	5.0 (1.5, 10.0)	6.0 (1.5, 11.0)	
NT-proBNP (pg/mL)					
N	533	544	544	542	
Mean (SD)	252.8 (662.7)	267.3 (1528.5)	181.1 (301.6)	266.2 (952.0)	0.8065
Median (IRQ)	78.0 (29.0, 216.0)	91.5 (36.5, 225.5)	78.0 (31.0, 187.0)	93.0 (34.0, 200.0)	

Values are n (%), mean (standard deviation), or median (interquartile range)

ESM Table 5 Baseline characteristics classified by quarters of the leucine distribution

	Leucine (mmol\L)				
**Characteristics**	**1st(<=0.069) (N = 896)**	**2nd(0.069 to <=0.080) (N = 897)**	**3rd(0.080 to <=0.092) (N = 895)**	**4th(>0.092) (N = 895)**	**p-value for trend**
Sex					
Male	434/ 896 (48.4%)	502/ 897 (56.0%)	579/ 895 (64.7%)	653/ 895 (73.0%)	<0.001
Female	462/ 896 (51.6%)	395/ 897 (44.0%)	316/ 895 (35.3%)	242/ 895 (27.0%)	
Current smokers					
No	794/ 896 (88.6%)	767/ 897 (85.5%)	745/ 895 (83.2%)	746/ 895 (83.4%)	<0.001
Yes	102/ 896 (11.4%)	130/ 897 (14.5%)	150/ 895 (16.8%)	149/ 895 (16.6%)	
History of macrovascular event					
No	612/ 896 (68.3%)	576/ 897 (64.2%)	589/ 895 (65.8%)	555/ 895 (62.0%)	0.0153
Yes	284/ 896 (31.7%)	321/ 897 (35.8%)	306/ 895 (34.2%)	340/ 895 (38.0%)	
Age (years)					
N	896	897	895	895	
Mean (SD)	68.0 (6.8)	67.5 (6.5)	66.8 (6.4)	65.0 (6.3)	<0.001
Median (IRQ)	69.0 (63.5, 73.0)	68.0 (63.0, 72.0)	67.0 (62.0, 71.0)	65.0 (60.0, 70.0)	
Duration of diabetes (years)					
N	896	897	894	895	
Mean (SD)	8.2 (7.2)	7.9 (6.4)	7.5 (6.2)	8.0 (6.0)	0.2179
Median (IRQ)	6.0 (3.0, 11.0)	7.0 (3.0, 11.0)	6.0 (2.0, 11.0)	7.0 (3.0, 12.0)	
BMI (Kg/m2)					
N	896	897	893	895	
Mean (SD)	28.9 (5.3)	30.1 (5.5)	30.3 (5.2)	30.7 (4.8)	<0.001

	Leucine (mmol\L)				
**Characteristics**	**1st(<=0.069)<br>(N = 896)**	**2nd(0.069 to <=0.080)<br>(N = 897)**	**3rd(0.080 to <=0.092)<br>(N = 895)**	**4th(>0.092)<br>(N = 895)**	**p-value<br>for trend**
Median (IRQ)	28.2 (25.4, 31.6)	29.4 (26.3, 33.2)	29.7 (26.7, 33.0)	30.1 (27.4, 33.3)	
History of heart failure					
No	854/ 896 (95.3%)	850/ 897 (94.8%)	861/ 895 (96.2%)	852/ 895 (95.2%)	0.7284
Yes	42/ 896 (4.7%)	47/ 897 (5.2%)	34/ 895 (3.8%)	43/ 895 (4.8%)	
Participation in moderate or vigorous activity					
No	483/ 896 (53.9%)	438/ 897 (48.8%)	480/ 895 (53.6%)	447/ 895 (49.9%)	0.3431
Yes	413/ 896 (46.1%)	459/ 897 (51.2%)	415/ 895 (46.4%)	448/ 895 (50.1%)	
SBP (mmHg)					
N	896	896	895	895	
Mean (SD)	146.6 (22.6)	147.8 (21.6)	146.8 (21.0)	147.5 (20.7)	0.6098
Median (IRQ)	144.5 (130.8, 159.0)	146.0 (133.0, 160.0)	144.5 (133.0, 158.0)	146.5 (133.0, 160.0)	
DBP (mmHg)					
N	896	896	895	895	
Mean (SD)	80.1 (10.7)	81.8 (11.1)	81.7 (10.7)	82.8 (10.7)	<0.001
Median (IRQ)	80.0 (72.5, 87.0)	81.5 (74.8, 88.5)	81.0 (74.5, 88.5)	82.5 (75.0, 90.0)	
Total chol (mmol/l)					
N	896	897	894	894	
Mean (SD)	5.1 (1.1)	5.1 (1.1)	5.1 (1.1)	5.2 (1.3)	0.0765
Median (IRQ)	5.0 (4.3, 5.9)	5.0 (4.3, 5.8)	5.0 (4.3, 5.8)	5.1 (4.3, 5.9)	
HDL Chol (mmol/l)					
N	895	897	893	893	

	Leucine (mmol\L)				
**Characteristics**	**1st(<=0.069) (N = 896)**	**2nd(0.069 to <=0.080) (N = 897)**	**3rd(0.080 to <=0.092) (N = 895)**	**4th(>0.092) (N = 895)**	**p-value for trend**
Mean (SD)	1.3 (0.4)	1.3 (0.3)	1.2 (0.3)	1.1 (0.3)	<0.001
Median (IRQ)	1.3 (1.0, 1.5)	1.2 (1.0, 1.4)	1.2 (1.0, 1.4)	1.1 (0.9, 1.3)	
Triacylglycerol (mmol/l)					
N	896	897	894	891	
Mean (SD)	1.4 (0.7)	1.7 (0.7)	2.0 (1.0)	2.7 (1.8)	<0.001
Median (IRQ)	1.3 (1.0, 1.7)	1.5 (1.2, 2.0)	1.8 (1.4, 2.4)	2.3 (1.6, 3.2)	
HbA1c (%)					
N	894	892	892	889	
Mean % (SD) [mmol/mol]	7.2 (1.4) [55]	7.3 (1.4) [56]	7.4 (1.4) [57]	7.7 (1.5) 61]	<0.001
Urinary albumin:creatinine ratio (mg/mmol)					
N	843	850	839	836	
Median (IRQ)	1.8 (0.8, 4.9)	1.9 (0.8, 5.1)	2 (0.9, 5.3)	2 (0.9, 6.3)	0.7714
Glucose (mmol/l)					
N	896	897	894	894	
Mean (SD)	7.9 (2.5)	8.1 (2.5)	8.5 (2.6)	9.3 (3.0)	<0.001
Median (IRQ)	7.5 (6.3, 8.9)	7.7 (6.4, 9.2)	8.0 (6.7, 9.8)	8.8 (7.2, 10.8)	
eGFR (mL/min/1.73 m²)					
N	894	892	892	888	
Mean (SD)	70.2 (17.2)	71.1 (16.7)	72.6 (16.5)	73.4 (16.3)	<0.001
Median (IRQ)	70.3 (57.9, 83.6)	71.8 (59.7, 84.7)	72.5 (62.0, 86.0)	73.7 (62.3, 86.2)	

	Leucine (mmol\L)				
**Characteristics**	**1st(<=0.069) (N = 896)**	**2nd(0.069 to <=0.080) (N = 897)**	**3rd(0.080 to <=0.092) (N = 895)**	**4th(>0.092) (N = 895)**	**p-value for trend**
CRP (nmol/l)					
N	872	871	876	872	
Mean (SD)	37.1 (84.8)	33.3 (61.0)	32.4 (48.6)	36.2 (69.5)	0.6817
Median (IRQ)	15.2 (7.6, 38.1)	18.1 (8.6, 39.0)	17.1 (8.6, 39.0)	19.0 (8.6, 41.0)	
hs-cTnT (ng/l)					
N	871	871	874	872	
Mean (SD)	9.0 (12.7)	8.8 (13.0)	8.4 (26.9)	9.3 (23.0)	0.9453
Median (IRQ)	5.0 (1.5, 11.0)	5.0 (1.5, 12.0)	5.0 (1.5, 10.0)	5.0 (1.5, 11.0)	
NT-proBNP (pg/mL)					
N	871	871	874	872	
Mean (SD)	391.2 (1564.3)	268.2 (554.7)	190.1 (402.4)	164.9 (307.3)	<0.001
Median (IRQ)	117.0 (51.0, 286.0)	100.0 (38.0, 229.0)	74.0 (31.0, 186.0)	65.0 (22.0, 164.0)	

Values are n (%), mean (standard deviation), or median (interquartile range)

ESM Table 6 Baseline characteristics classified by quarters of the alanine distribution

Characteristics	Alanine (mmol\L) 1st(<=0.326) (N = 897)	2nd(0.326 to <=0.365) (N = 897)	3rd(0.365 to <=0.407) (N = 897)	4th(>0.407) (N = 895)	p-value for trend
Sex					
Male	591/ 897 (65.9%)	565/ 897 (63.0%)	511/ 897 (57.0%)	503/ 895 (56.2%)	<0.001
Female	306/ 897 (34.1%)	332/ 897 (37.0%)	386/ 897 (43.0%)	392/ 895 (43.8%)	
Current smokers					
No	760/ 897 (84.7%)	773/ 897 (86.2%)	738/ 897 (82.3%)	783/ 895 (87.5%)	0.4112
Yes	137/ 897 (15.3%)	124/ 897 (13.8%)	159/ 897 (17.7%)	112/ 895 (12.5%)	
History of macrovascular event					
No	613/ 897 (68.3%)	589/ 897 (65.7%)	600/ 897 (66.9%)	532/ 895 (59.4%)	<0.001
Yes	284/ 897 (31.7%)	308/ 897 (34.3%)	297/ 897 (33.1%)	363/ 895 (40.6%)	
Age (years)					
N	897	897	897	895	
Mean (SD)	67.5 (6.8)	67.4 (6.3)	66.2 (6.5)	66.2 (6.5)	<0.001
Median (IRQ)	68.0 (63.0, 72.0)	67.0 (63.0, 72.0)	66.0 (61.0, 71.0)	67.0 (61.0, 71.0)	
Duration of diabetes (years)					
N	896	897	897	895	
Mean (SD)	8.4 (6.9)	7.7 (6.4)	7.4 (6.3)	8.2 (6.3)	0.2860
Median (IRQ)	7.0 (3.0, 12.0)	6.0 (3.0, 11.0)	6.0 (2.0, 11.0)	7.0 (3.0, 12.0)	
BMI (Kg/m2)					
N	897	896	896	895	
Mean (SD)	29.2 (5.1)	29.9 (5.3)	30.6 (5.6)	30.3 (4.9)	<0.001

Characteristics	Alanine (mmol\L)<br>1st(<=0.326)<br>(N = 897)	2nd(0.326 to <=0.365)<br>(N = 897)	3rd(0.365 to <=0.407)<br>(N = 897)	4th(>0.407)<br>(N = 895)	p-value<br>for trend
Median (IRQ)	28.6 (25.7, 32.2)	29.1 (26.3, 32.8)	29.7 (26.8, 33.3)	29.7 (27.0, 32.8)	
History of heart failure					
No	857/ 897 (95.5%)	856/ 897 (95.4%)	853/ 897 (95.1%)	854/ 895 (95.4%)	0.8234
Yes	40/ 897 (4.5%)	41/ 897 (4.6%)	44/ 897 (4.9%)	41/ 895 (4.6%)	
Participation in moderate or vigorous activity					
No	479/ 897 (53.4%)	457/ 897 (50.9%)	452/ 897 (50.4%)	462/ 895 (51.6%)	0.4291
Yes	418/ 897 (46.6%)	440/ 897 (49.1%)	445/ 897 (49.6%)	433/ 895 (48.4%)	
SBP (mmHg)					
N	897	896	897	895	
Mean (SD)	146.7 (21.4)	147.5 (21.6)	147.1 (21.0)	147.4 (22.0)	0.5705
Median (IRQ)	145.0 (131.5, 159.5)	145.0 (133.0, 158.5)	145.0 (133.0, 160.0)	145.0 (132.0, 160.0)	
DBP (mmHg)					
N	897	896	897	895	
Mean (SD)	80.8 (10.8)	81.4 (10.6)	81.7 (10.6)	82.5 (11.4)	0.0014
Median (IRQ)	80.0 (73.5, 88.0)	80.8 (73.8, 88.5)	81.5 (75.0, 88.5)	82.0 (74.5, 90.0)	
Total chol (mmol/l)					
N	896	897	897	894	
Mean (SD)	5.0 (1.1)	5.1 (1.1)	5.2 (1.3)	5.2 (1.2)	0.0012
Median (IRQ)	4.9 (4.2, 5.7)	5.1 (4.3, 5.9)	5.1 (4.4, 5.9)	5.1 (4.4, 5.9)	
HDL Chol (mmol/l)					
N	895	897	896	893	

Characteristics	Alanine (mmol\L)<br>1st(<=0.326)<br>(N = 897)	2nd(0.326 to <=0.365)<br>(N = 897)	3rd(0.365 to <=0.407)<br>(N = 897)	4th(>0.407)<br>(N = 895)	p-value<br>for trend
Mean (SD)	1.2 (0.3)	1.2 (0.3)	1.2 (0.3)	1.2 (0.3)	0.8507
Median (IRQ)	1.2 (1.0, 1.4)	1.2 (1.0, 1.4)	1.2 (1.0, 1.4)	1.2 (1.0, 1.4)	
Triacylglycerol (mmol/l)					
N	896	896	896	893	
Mean (SD)	1.6 (1.0)	1.9 (1.1)	2.1 (1.3)	2.3 (1.4)	<0.001
Median (IRQ)	1.4 (1.0, 1.8)	1.6 (1.2, 2.3)	1.8 (1.4, 2.5)	1.9 (1.5, 2.7)	
HbA1c (%)					
N	893	892	893	892	
Mean % (SD) [mmol/mol]	7.3 (1.5) [56]	7.4 (1.5) [57]	7.4 (1.4) [57]	7.5 (1.4) [59]	0.0175
Urinary albumin:creatinine ratio (mg/mmol)					
N	842	851	833	845	
Median (IRQ)	1.8 (0.8, 5.5)	1.9 (0.8, 4.9)	1.9 (0.8, 4.8)	2.1 (0.9, 6)	0.1522
Glucose (mmol/l)					
N	897	897	896	894	
Mean (SD)	8.1 (2.7)	8.4 (2.7)	8.5 (2.7)	8.9 (2.7)	<0.001
Median (IRQ)	7.6 (6.4, 9.3)	7.9 (6.6, 9.6)	8.0 (6.7, 9.7)	8.3 (7.0, 10.3)	
eGFR (mL/min/1.73 m^2)					
N	893	894	891	891	
Mean (SD)	71.7 (16.8)	71.3 (17.0)	72.1 (16.9)	72.1 (16.3)	0.4152
Median (IRQ)	72.2 (59.4, 85.0)	71.5 (60.4, 84.2)	72.9 (61.1, 85.3)	71.7 (60.1, 85.4)	

	Alanine (mmol\L)				
**Characteristics**	**1st(<=0.326) (N = 897)**	**2nd(0.326 to <=0.365) (N = 897)**	**3rd(0.365 to <=0.407) (N = 897)**	**4th(>0.407) (N = 895)**	**p-value for trend**
CRP (nmol/l)					
N	877	871	874	869	
Mean (SD)	49.5 (109.5)	32.4 (43.8)	32.4 (48.6)	25.7 (39.0)	<0.001
Median (IRQ)	19.0 (8.6, 49.5)	16.2 (8.6, 39.0)	19.0 (8.6, 41.0)	15.2 (7.6, 30.5)	
hs-cTnT (ng/l)					
N	876	871	872	869	
Mean (SD)	10.4 (29.0)	9.3 (18.4)	8.0 (17.0)	7.8 (10.3)	0.0025
Median (IRQ)	5.0 (1.5, 12.0)	5.0 (1.5, 12.0)	5.0 (1.5, 10.0)	5.0 (1.5, 10.0)	
NT-proBNP (pg/mL)					
N	876	871	872	869	
Mean (SD)	306.6 (789.9)	320.6 (1458.9)	186.7 (367.0)	199.9 (371.1)	<0.001
Median (IRQ)	105.0 (42.0, 259.0)	90.0 (37.0, 246.0)	73.5 (32.0, 168.5)	85.0 (28.0, 203.0)	

Values are n (%), mean (standard deviation), or median (interquartile range)

ESM Table 7 Baseline characteristics classified by quarters of the tyrosine distribution

	Tyrosine (mmol\L)				
**Characteristics**	**1st(<=0.045) (N = 897)**	**2nd(0.045 to <=0.052) (N = 893)**	**3rd(0.052 to <=0.059) (N = 895)**	**4th(>0.059) (N = 894)**	**p-value for trend**
Sex					
Male	521/ 897 (58.1%)	547/ 893 (61.3%)	512/ 895 (57.2%)	586/ 894 (65.5%)	0.0121
Female	376/ 897 (41.9%)	346/ 893 (38.7%)	383/ 895 (42.8%)	308/ 894 (34.5%)	
Current smokers					
No	763/ 897 (85.1%)	761/ 893 (85.2%)	764/ 895 (85.4%)	760/ 894 (85.0%)	0.99
Yes	134/ 897 (14.9%)	132/ 893 (14.8%)	131/ 895 (14.6%)	134/ 894 (15.0%)	
History of macrovascular event					
No	616/ 897 (68.7%)	574/ 893 (64.3%)	582/ 895 (65.0%)	557/ 894 (62.3%)	0.0100
Yes	281/ 897 (31.3%)	319/ 893 (35.7%)	313/ 895 (35.0%)	337/ 894 (37.7%)	
Age (years)					
N	897	893	895	894	
Mean (SD)	67.3 (6.7)	67.1 (6.6)	66.4 (6.5)	66.6 (6.6)	0.0042
Median (IRQ)	67.0 (62.0, 72.0)	67.0 (62.0, 72.0)	67.0 (61.0, 71.0)	66.0 (61.0, 71.0)	
Duration of diabetes (years)					
N	897	892	895	894	
Mean (SD)	9.4 (6.9)	8.2 (6.6)	7.1 (6.0)	6.9 (5.9)	<0.001
Median (IRQ)	9.0 (4.0, 14.0)	7.0 (3.0, 12.0)	5.0 (2.0, 10.0)	5.0 (2.0, 10.0)	
BMI (Kg/m2)					
N	897	891	895	894	
Mean (SD)	29.0 (5.3)	29.7 (5.1)	30.5 (5.3)	30.8 (5.2)	<0.001

	Tyrosine (mmol\L)				
**Characteristics**	**1st(<=0.045) (N = 897)**	**2nd(0.045 to <=0.052) (N = 893)**	**3rd(0.052 to <=0.059) (N = 895)**	**4th(>0.059) (N = 894)**	**p-value for trend**
Median (IRQ)	28.4 (25.3, 31.8)	29.0 (26.3, 32.3)	29.7 (27.0, 33.2)	30.1 (27.2, 33.7)	
History of heart failure					
No	866/ 897 (96.5%)	855/ 893 (95.7%)	850/ 895 (95.0%)	842/ 894 (94.2%)	0.0128
Yes	31/ 897 (3.5%)	38/ 893 (4.3%)	45/ 895 (5.0%)	52/ 894 (5.8%)	
Participation in moderate or vigorous activity					
No	441/ 897 (49.2%)	477/ 893 (53.4%)	465/ 895 (52.0%)	467/ 894 (52.2%)	0.2980
Yes	456/ 897 (50.8%)	416/ 893 (46.6%)	430/ 895 (48.0%)	427/ 894 (47.8%)	
SBP (mmHg)					
N	897	893	894	894	
Mean (SD)	148.2 (22.8)	147.8 (21.4)	146.4 (21.0)	146.2 (20.7)	0.0221
Median (IRQ)	145.5 (132.0, 161.5)	146.0 (132.5, 160.0)	145.0 (132.0, 158.0)	145.0 (132.5, 157.5)	
DBP (mmHg)					
N	897	893	894	894	
Mean (SD)	80.9 (11.0)	81.4 (10.6)	82.2 (11.0)	81.9 (10.8)	0.0160
Median (IRQ)	80.0 (73.5, 88.0)	81.0 (74.0, 88.5)	82.0 (75.0, 89.0)	81.5 (74.5, 89.0)	
Total chol (mmol/l)					
N	897	892	895	893	
Mean (SD)	5.2 (1.1)	5.1 (1.1)	5.3 (1.3)	5.0 (1.1)	0.1502
Median (IRQ)	5.1 (4.3, 5.9)	5.0 (4.3, 5.9)	5.1 (4.4, 5.9)	4.9 (4.3, 5.7)	
HDL Chol (mmol/l)					
N	897	891	894	892	

	Tyrosine (mmol\L)				
**Characteristics**	**1st(<=0.045) (N = 897)**	**2nd(0.045 to <=0.052) (N = 893)**	**3rd(0.052 to <=0.059) (N = 895)**	**4th(>0.059) (N = 894)**	**p-value for trend**
Mean (SD)	1.2 (0.3)	1.2 (0.3)	1.2 (0.3)	1.2 (0.3)	0.0072
Median (IRQ)	1.2 (1.0, 1.4)	1.2 (1.0, 1.4)	1.2 (1.0, 1.4)	1.2 (1.0, 1.4)	
Triacylglycerol (mmol/l)					
N	896	892	894	892	
Mean (SD)	1.9 (1.1)	1.9 (1.2)	2.0 (1.4)	2.0 (1.2)	0.0446
Median (IRQ)	1.6 (1.2, 2.3)	1.6 (1.2, 2.3)	1.8 (1.3, 2.4)	1.7 (1.3, 2.4)	
HbA1c (%)					
N	894	891	890	888	
Mean % (SD) [mmol/mol]	7.6 (1.6) [60]	7.4 (1.4) [57]	7.3 (1.4) [56]	7.3 (1.3) [56]	<0.001
Urinary albumin:creatinine ratio (mg/mmol)					
N	844	839	841	840	
Median (IRQ)	2.4 (1.1, 7.1)	2 (0.9, 5.5)	1.6 (0.8, 4.3)	1.6 (0.8, 4.4)	<0.001
Glucose (mmol/l)					
N	896	893	895	893	
Mean (SD)	8.6 (2.9)	8.5 (2.6)	8.4 (2.8)	8.3 (2.4)	0.0152
Median (IRQ)	8.0 (6.6, 10.0)	8.0 (6.6, 9.7)	7.8 (6.6, 9.7)	7.9 (6.6, 9.6)	
eGFR (mL/min/1.73 m²)					
N	894	889	890	889	
Mean (SD)	70.9 (17.7)	70.9 (17.1)	72.4 (16.2)	73.1 (15.6)	0.0011
Median (IRQ)	71.9 (58.1, 85.1)	71.2 (59.1, 84.4)	72.8 (61.6, 84.9)	72.2 (62.3, 86.0)	

Characteristics	Tyrosine (mmol\L) 1st(<=0.045) (N = 897)	2nd(0.045 to <=0.052) (N = 893)	3rd(0.052 to <=0.059) (N = 895)	4th(>0.059) (N = 894)	p-value for trend
CRP (nmol/l)					
N	881	867	868	867	
Mean (SD)	35.2 (81.0)	32.4 (54.3)	34.3 (56.2)	37.1 (73.3)	0.5176
Median (IRQ)	15.2 (7.6, 34.3)	16.2 (8.6, 37.1)	19.0 (9.5, 41.9)	19.0 (8.6, 41.9)	
hs-cTnT (ng/l)					
N	880	866	868	866	
Mean (SD)	9.4 (12.9)	8.9 (26.9)	8.1 (13.1)	9.2 (23.0)	0.6502
Median (IRQ)	5.0 (1.5, 12.0)	5.0 (1.5, 11.0)	5.0 (1.5, 10.0)	5.0 (1.5, 11.0)	
NT-proBNP (pg/mL)					
N	880	866	868	866	
Mean (SD)	318.3 (1443.2)	256.6 (562.7)	217.2 (627.6)	220.9 (474.8)	0.0119
Median (IRQ)	103.0 (42.0, 246.0)	90.0 (36.0, 222.0)	76.0 (28.0, 191.0)	79.0 (31.0, 200.0)	

Values are n (%), mean (standard deviation), or median (interquartile range)

ESM Table 8 Baseline characteristics classified by quarters of the histidine distribution

	Histidine (mmol\L)				
**Characteristics**	**1st(<=0.044) (N = 892)**	**2nd(0.044 to <=0.050) (N = 891)**	**3rd(0.050 to <=0.056) (N = 893)**	**4th(>0.056) (N = 890)**	**p-value for trend**
Sex					
Male	444/ 892 (49.8%)	534/ 891 (59.9%)	565/ 893 (63.3%)	617/ 890 (69.3%)	<0.001
Female	448/ 892 (50.2%)	357/ 891 (40.1%)	328/ 893 (36.7%)	273/ 890 (30.7%)	
Current smokers					
No	756/ 892 (84.8%)	753/ 891 (84.5%)	764/ 893 (85.6%)	765/ 890 (86.0%)	0.3826
Yes	136/ 892 (15.2%)	138/ 891 (15.5%)	129/ 893 (14.4%)	125/ 890 (14.0%)	
History of macrovascular event					
No	581/ 892 (65.1%)	599/ 891 (67.2%)	585/ 893 (65.5%)	556/ 890 (62.5%)	0.1747
Yes	311/ 892 (34.9%)	292/ 891 (32.8%)	308/ 893 (34.5%)	334/ 890 (37.5%)	
Age (years)					
N	892	891	893	890	
Mean (SD)	67.2 (6.6)	66.9 (6.6)	67.0 (6.6)	66.2 (6.5)	0.0019
Median (IRQ)	68.0 (62.0, 72.0)	67.0 (62.0, 72.0)	67.0 (62.0, 71.0)	66.0 (61.0, 71.0)	
Duration of diabetes (years)					
N	892	891	892	890	
Mean (SD)	8.1 (6.6)	7.9 (6.5)	8.1 (6.6)	7.6 (6.2)	0.1189
Median (IRQ)	7.0 (3.0, 11.0)	6.0 (3.0, 11.0)	6.0 (3.0, 12.0)	6.0 (3.0, 11.0)	
BMI (Kg/m2)					
N	892	889	893	890	
Mean (SD)	30.2 (5.4)	30.3 (5.4)	29.8 (4.9)	29.7 (5.3)	0.0066

	Histidine (mmol\L)				
**Characteristics**	**1st(<=0.044) (N = 892)**	**2nd(0.044 to <=0.050) (N = 891)**	**3rd(0.050 to <=0.056) (N = 893)**	**4th(>0.056) (N = 890)**	**p-value for trend**
Median (IRQ)	29.6 (26.6, 33.3)	29.7 (26.6, 33.2)	29.1 (26.5, 32.4)	29.0 (26.3, 32.1)	
History of heart failure					
No	829/ 892 (92.9%)	854/ 891 (95.8%)	857/ 893 (96.0%)	860/ 890 (96.6%)	<0.001
Yes	63/ 892 (7.1%)	37/ 891 (4.2%)	36/ 893 (4.0%)	30/ 890 (3.4%)	
Participation in moderate or vigorous activity					
No	514/ 892 (57.6%)	472/ 891 (53.0%)	443/ 893 (49.6%)	415/ 890 (46.6%)	<0.001
Yes	378/ 892 (42.4%)	419/ 891 (47.0%)	450/ 893 (50.4%)	475/ 890 (53.4%)	
SBP (mmHg)					
N	892	890	893	890	
Mean (SD)	146.5 (21.7)	146.3 (20.9)	147.6 (21.7)	148.0 (21.3)	0.0645
Median (IRQ)	144.5 (131.3, 159.5)	144.5 (132.0, 158.5)	146.0 (133.5, 159.0)	145.5 (132.5, 160.5)	
DBP (mmHg)					
N	892	890	893	890	
Mean (SD)	80.9 (10.8)	81.4 (10.9)	81.8 (10.9)	82.1 (10.6)	0.0163
Median (IRQ)	80.0 (73.5, 88.3)	81.0 (74.0, 89.0)	81.5 (75.0, 88.5)	82.0 (74.5, 89.0)	
Total chol (mmol/l)					
N	891	891	893	889	
Mean (SD)	5.2 (1.1)	5.2 (1.1)	5.1 (1.1)	5.1 (1.3)	0.4220
Median (IRQ)	5.1 (4.3, 5.9)	5.1 (4.3, 5.9)	5.0 (4.3, 5.9)	5.0 (4.3, 5.8)	
HDL Chol (mmol/l)					
N	889	891	893	888	

Characteristics	Histidine (mmol\L)<br>1st(<=0.044)<br>(N = 892)	2nd(0.044 to <=0.050)<br>(N = 891)	3rd(0.050 to <=0.056)<br>(N = 893)	4th(>0.056)<br>(N = 890)	p-value<br>for trend
Mean (SD)	1.2 (0.3)	1.2 (0.3)	1.2 (0.3)	1.2 (0.3)	0.8696
Median (IRQ)	1.2 (1.0, 1.4)	1.2 (1.0, 1.4)	1.2 (1.0, 1.4)	1.2 (1.0, 1.4)	
Triacylglycerol (mmol/l)					
N	889	890	893	889	
Mean (SD)	2.0 (1.1)	2.0 (1.2)	2.0 (1.3)	1.9 (1.2)	0.8676
Median (IRQ)	1.7 (1.2, 2.4)	1.7 (1.2, 2.4)	1.7 (1.3, 2.3)	1.6 (1.2, 2.3)	
HbA1c (%)					
N	888	885	889	888	
Mean % (SD) [mmol/mol]	7.7 (1.7) [61]	7.5 (1.4) [59]	7.3 (1.3) [56]	7.1 (1.3) [54]	<0.001
Urinary albumin:creatinine ratio (mg/mmol)					
N	844	831	844	833	
Median (IRQ)	2.2 (1.1, 7)	2 (0.8, 5.3)	1.8 (0.8, 4.7)	1.6 (0.7, 4.5)	0.0161
Glucose (mmol/l)					
N	892	891	892	889	
Mean (SD)	8.8 (3.0)	8.6 (2.8)	8.3 (2.6)	8.1 (2.4)	<0.001
Median (IRQ)	8.2 (6.7, 10.3)	8.0 (6.7, 9.8)	7.9 (6.5, 9.4)	7.6 (6.5, 9.2)	
eGFR (mL/min/1.73 m²)					
N	890	887	888	884	
Mean (SD)	70.6 (16.9)	71.7 (16.7)	72.4 (16.0)	72.5 (17.1)	0.0106
Median (IRQ)	70.1 (57.6, 83.7)	71.8 (60.0, 84.8)	72.9 (61.6, 85.3)	73.3 (61.0, 85.7)	

	Histidine (mmol\L)				
**Characteristics**	**1st(<=0.044) (N = 892)**	**2nd(0.044 to <=0.050) (N = 891)**	**3rd(0.050 to <=0.056) (N = 893)**	**4th(>0.056) (N = 890)**	**p-value for trend**
CRP (nmol/l)					
N	870	872	872	866	
Mean (SD)	51.4 (98.1)	34.3 (51.4)	30.5 (51.4)	24.8 (53.3)	<0.001
Median (IRQ)	23.8 (10.5, 56.2)	19.0 (9.5, 40.0)	16.2 (7.6, 34.3)	12.4 (6.7, 27.6)	
hs-cTnT (ng/l)					
N	870	870	872	865	
Mean (SD)	9.6 (31.4)	8.8 (11.3)	8.4 (12.6)	8.8 (17.6)	0.3204
Median (IRQ)	4.0 (1.5, 11.0)	5.0 (1.5, 11.0)	5.0 (1.5, 11.0)	6.0 (1.5, 11.0)	
NT-proBNP (pg/mL)					
N	870	870	872	865	
Mean (SD)	312.6 (785.2)	281.4 (1302.1)	203.6 (426.7)	216.8 (739.2)	0.0058
Median (IRQ)	100.0 (37.0, 268.0)	96.0 (40.0, 223.0)	81.5 (30.5, 192.5)	75.0 (31.0, 193.0)	

Values are n (%), mean (standard deviation), or median (interquartile range)

ESM Table 9 Baseline characteristics classified by quarters of the valine distribution

Characteristics	Valine (mmol\L) 1st(<=0.150) (N = 897)	2nd(0.150 to <=0.173) (N = 901)	3rd(0.173 to <=0.196) (N = 895)	4th(>0.196) (N = 894)	p-value for trend
Sex					
Male	432/ 897 (48.2%)	514/ 901 (57.0%)	593/ 895 (66.3%)	631/ 894 (70.6%)	<0.001
Female	465/ 897 (51.8%)	387/ 901 (43.0%)	302/ 895 (33.7%)	263/ 894 (29.4%)	
Current smokers					
No	755/ 897 (84.2%)	780/ 901 (86.6%)	761/ 895 (85.0%)	758/ 894 (84.8%)	0.9536
Yes	142/ 897 (15.8%)	121/ 901 (13.4%)	134/ 895 (15.0%)	136/ 894 (15.2%)	
History of macrovascular event					
No	612/ 897 (68.2%)	582/ 901 (64.6%)	590/ 895 (65.9%)	551/ 894 (61.6%)	0.0096
Yes	285/ 897 (31.8%)	319/ 901 (35.4%)	305/ 895 (34.1%)	343/ 894 (38.4%)	
Age (years)					
N	897	901	895	894	
Mean (SD)	67.9 (6.9)	67.1 (6.6)	66.9 (6.3)	65.4 (6.3)	<0.001
Median (IRQ)	69.0 (63.0, 73.0)	67.0 (62.0, 72.0)	67.0 (62.0, 71.0)	66.0 (60.0, 70.0)	
Duration of diabetes (years)					
N	897	901	894	894	
Mean (SD)	8.1 (7.0)	8.1 (6.7)	7.5 (6.0)	7.9 (6.1)	0.1826
Median (IRQ)	6.0 (3.0, 12.0)	7.0 (3.0, 11.0)	6.0 (2.0, 11.0)	6.0 (3.0, 12.0)	
BMI (Kg/m2)					
N	897	899	895	894	
Mean (SD)	29.0 (5.2)	29.9 (5.2)	30.3 (5.4)	30.8 (5.1)	<0.001
Median (IRQ)	28.4 (25.5, 31.6)	29.1 (26.6, 32.7)	29.4 (26.7, 33.0)	30.3 (27.3, 33.6)	

Characteristics	Valine (mmol\L)<br>1st(<=0.150)<br>(N = 897)	2nd(0.150 to <=0.173)<br>(N = 901)	3rd(0.173 to <=0.196)<br>(N = 895)	4th(>0.196)<br>(N = 894)	p-value<br>for trend
History of heart failure					
No	851/ 897 (94.9%)	852/ 901 (94.6%)	861/ 895 (96.2%)	857/ 894 (95.9%)	0.1424
Yes	46/ 897 (5.1%)	49/ 901 (5.4%)	34/ 895 (3.8%)	37/ 894 (4.1%)	
Participation in moderate or vigorous activity					
No	484/ 897 (54.0%)	472/ 901 (52.4%)	446/ 895 (49.8%)	449/ 894 (50.2%)	0.0656
Yes	413/ 897 (46.0%)	429/ 901 (47.6%)	449/ 895 (50.2%)	445/ 894 (49.8%)	
SBP (mmHg)					
N	897	900	895	894	
Mean (SD)	147.1 (22.4)	147.3 (21.7)	147.6 (21.1)	146.8 (20.8)	0.9113
Median (IRQ)	145.5 (131.5, 160.0)	145.0 (132.5, 159.0)	145.0 (133.0, 160.0)	145.0 (133.0, 159.0)	
DBP (mmHg)					
N	897	900	895	894	
Mean (SD)	80.9 (11.2)	81.2 (10.8)	82.1 (10.5)	82.3 (10.9)	0.0011
Median (IRQ)	80.0 (72.5, 88.5)	80.3 (74.0, 87.8)	81.5 (75.0, 88.5)	82.0 (75.0, 89.5)	
Total chol (mmol/l)					
N	897	901	895	892	
Mean (SD)	5.3 (1.2)	5.2 (1.1)	5.0 (1.1)	5.1 (1.3)	<0.001
Median (IRQ)	5.2 (4.5, 6.1)	5.1 (4.4, 5.9)	5.0 (4.3, 5.8)	4.9 (4.3, 5.7)	
HDL Chol (mmol/l)					
N	896	900	894	892	
Mean (SD)	1.3 (0.4)	1.2 (0.3)	1.2 (0.3)	1.2 (0.3)	<0.001

Characteristics	Valine (mmol\L)<br>1st(<=0.150)<br>(N = 897)	2nd(0.150 to <=0.173)<br>(N = 901)	3rd(0.173 to <=0.196)<br>(N = 895)	4th(>0.196)<br>(N = 894)	p-value<br>for trend
Median (IRQ)	1.3 (1.1, 1.5)	1.2 (1.0, 1.4)	1.2 (1.0, 1.4)	1.1 (1.0, 1.3)	
Triacylglycerol (mmol/l)					
N	896	901	895	890	
Mean (SD)	1.8 (1.3)	1.9 (1.0)	2.0 (1.2)	2.2 (1.3)	<0.001
Median (IRQ)	1.5 (1.1, 2.1)	1.6 (1.2, 2.3)	1.7 (1.2, 2.3)	1.9 (1.4, 2.6)	
HbA1c (%)					
N	893	898	891	889	
Mean % (SD) [mmol/mol]	7.2 (1.5) [55]	7.3 (1.4) [56]	7.4 (1.3) [57]	7.7 (1.5) [61]	<0.001
Urinary albumin:creatinine ratio (mg/mmol)					
N	836	844	854	838	
Median (IRQ)	1.9 (0.8, 5.3)	2.1 (0.9, 5.9)	1.8 (0.8, 4.5)	1.9 (0.8, 5.9)	0.0028
Glucose (mmol/l)					
N	897	901	894	893	
Mean (SD)	8.0 (2.8)	8.2 (2.5)	8.5 (2.5)	9.2 (2.9)	<0.001
Median (IRQ)	7.4 (6.2, 9.1)	7.7 (6.5, 9.3)	8.0 (6.8, 9.6)	8.6 (7.1, 10.6)	
eGFR (mL/min/1.73 m²)					
N	894	895	895	886	
Mean (SD)	68.8 (17.8)	71.7 (16.7)	72.4 (16.0)	74.3 (15.8)	<0.001
Median (IRQ)	69.0 (56.7, 82.7)	71.7 (60.4, 84.4)	72.5 (61.4, 85.0)	74.3 (63.2, 87.3)	
CRP (nmol/l)					

Characteristics	Valine (mmol\L)<br>1st(<=0.150)<br>(N = 897)	2nd(0.150 to <=0.173)<br>(N = 901)	3rd(0.173 to <=0.196)<br>(N = 895)	4th(>0.196)<br>(N = 894)	p-value<br>for trend
N	877	872	878	864	
Mean (SD)	40.0 (88.6)	35.2 (61.0)	29.5 (48.6)	35.2 (64.8)	0.0619
Median (IRQ)	17.1 (7.6, 41.0)	18.1 (9.4, 40.0)	17.1 (8.6, 35.2)	18.1 (8.6, 41.0)	
hs-cTnT (ng/l)					
N	877	871	876	864	
Mean (SD)	9.7 (13.4)	9.7 (28.3)	7.5 (9.3)	8.7 (22.8)	0.0902
Median (IRQ)	5.0 (1.5, 12.0)	5.0 (1.5, 11.0)	5.0 (1.5, 10.0)	5.0 (1.5, 10.0)	
NT-proBNP (pg/mL)					
N	877	871	876	864	
Mean (SD)	377.0 (1443.8)	274.2 (835.6)	190.1 (328.3)	171.6 (334.4)	<0.001
Median (IRQ)	115.0 (49.0, 279.0)	97.0 (37.0, 221.0)	75.0 (28.0, 203.0)	66.0 (27.0, 167.0)	

Values are n (%), mean (standard deviation), or median (interquartile range)

ESM Table 10 Associations of the amino acids with risk of all 3 endpoints (per 1 standard deviation increase)

	Macrovascular events			Microvascular events			All Deaths		
	**HR**	**95% CI**	**p-value**	**HR**	**95% CI**	**p-value**	**HR**	**95% CI**	**p-value**
**Model 1**									
Phenylalanine (1SD=0.0088)	1.22	1.12,1.32	<0.001	1.09	0.98,1.22	0.110	1.21	1.12,1.32	<0.001
Isoleucine (1SD=0.0170)	1.06	0.98,1.15	0.152	1.09	0.99,1.2	0.064	1.00	0.91,1.1	0.950
Glutamine (1SD=0.1096)	0.88	0.79,0.98	0.020	0.87	0.76,1.01	0.062	0.85	0.76,0.94	0.002
Leucine (1SD=0.0197)	0.96	0.87,1.05	0.325	1.04	0.93,1.16	0.508	0.89	0.8,0.99	0.031
Alanine (1SD=0.0645)	0.96	0.88,1.05	0.385	0.95	0.85,1.07	0.425	0.91	0.83,1	0.040
Tyrosine (1SD=0.0112)	1.01	0.92,1.1	0.874	0.74	0.64,0.86	<0.001	0.96	0.87,1.06	0.418
Histidine (1SD=0.0095)	0.86	0.79,0.94	0.001	0.93	0.83,1.05	0.237	0.83	0.76,0.91	<0.001
Valine (1SD=0.0352)	0.94	0.86,1.03	0.167	1.00	0.89,1.13	0.970	0.81	0.73,0.89	<0.001
**Model 2**									
Phenylalanine (1SD=0.0088)	1.09	0.98,1.21	0.105	1.02	0.89,1.17	0.767	1.04	0.94,1.16	0.440
Isoleucine (1SD=0.0170)	1.01	0.90,1.13	0.905	0.93	0.80,1.08	0.321	0.90	0.79,1.02	0.112
Glutamine (1SD=0.1096)	0.93	0.83,1.05	0.261	0.91	0.78,1.06	0.229	0.93	0.83,1.05	0.262
Leucine (1SD=0.0197)	0.91	0.81,1.02	0.101	0.89	0.77,1.04	0.140	0.79	0.69,0.90	0.001
Alanine (1SD=0.0645)	0.98	0.89,1.08	0.704	0.86	0.76,0.98	0.022	0.91	0.83,1.01	0.075
Tyrosine (1SD=0.0112)	1.01	0.91,1.12	0.868	0.78	0.67,0.91	0.002	0.96	0.86,1.07	0.456
Histidine (1SD=0.0095)	0.92	0.84,1.01	0.089	0.92	0.82,1.03	0.147	0.89	0.81,0.99	0.024
Valine (1SD=0.0352)	0.94	0.84,1.04	0.247	0.91	0.80,1.04	0.150	0.79	0.70,0.88	<0.001

Model 1 adjusted for Age, sex, regions and randomised treatment

Model 2 additionally adjusted for previous macrovascular event, duration of diabetes, current smoking, systolic blood pressure, BMI (Kg/m2), albumin/creatinine ratio, estimated glomerular filtration rate, HbA1c, plasma Glucose (mmol/l), total and HDL Chol (mmol/l) cholesterol, Triacylglycerol (mmol/l), aspirin or other antiplatelet agent, statin or other lipid-lowering agent, β-

blocker, ACE inhibitor or angiotensin receptor blocker, metformin use, history of heart failure, participation in moderate and/or vigorous exercise for >15 min at least once weekly, and C-reactive protein.

ESM Table 11: Sensitivity analysis - Associations of the amino acids with risk of all 3 endpoints (per 1 standard deviation increase) in those with no missing values in model 2 of table s10

	Macrovascular events			Microvascular events			All Deaths		
	**HR**	**95% CI**	**p-value**	**HR**	**95% CI**	**p-value**	**HR**	**95% CI**	**p-value**
**Model 1 * (sensitivity)**									
Phenylalanine (1SD=0.0088)	1.19	1.09,1.30	<0.001	1.05	0.98,1.22	0.381	1.17	1.07,1.28	<0.001
Isoleucine (1SD=0.0170)	1.05	0.96,1.14	0.300	1.05	0.99,1.2	0.312	0.98	0.88,1.08	0.635
Glutamine (1SD=0.1096)	0.87	0.78,0.98	0.017	0.84	0.76,1.01	0.017	0.86	0.77,0.96	0.009
Leucine (1SD=0.0197)	0.95	0.86,1.05	0.321	1.01	0.93,1.16	0.868	0.87	0.78,0.98	0.017
Alanine (1SD=0.0645)	0.97	0.89,1.06	0.499	0.90	0.85,1.07	0.091	0.90	0.82,0.99	0.033
Tyrosine (1SD=0.0112)	0.99	0.89,1.09	0.766	0.70	0.64,0.86	<0.001	0.95	0.86,1.06	0.364
Histidine (1SD=0.0095)	0.86	0.79,0.95	0.002	0.89	0.83,1.05	0.044	0.83	0.76,0.92	<0.001
Valine (1SD=0.0352)	0.95	0.86,1.04	0.282	0.97	0.89,1.13	0.609	0.81	0.73,0.90	<0.001

Model 1 adjusted for Age, sex, regions and randomised treatment

References

1. Soininen P, Kangas AJ, Würtz P, Suna T, Ala-Korpela M. Quantitative serum nuclear magnetic resonance metabolomics in cardiovascular epidemiology and genetics. Circ Cardiovasc Genet. 2015;8:192–206.

2. Tillin T, Hughes AD, Wang Q, Würtz P, Ala-Korpela M, Sattar N, et al. Diabetes risk and amino acid profiles: cross-sectional and prospective analyses of ethnicity, amino acids and diabetes in a South Asian and European cohort from the SABRE (Southall And Brent REvisited) Study. Diabetologia. 2015;58:968–79.

3. Würtz P, Havulinna AS, Soininen P, Tynkkynen T, Prieto-Merino D, Tillin T, et al. Metabolite profiling and cardiovascular event risk: a prospective study of 3 population-based cohorts. Circulation. 2015;131:774–85.

4. Kaikkonen JE, Würtz P, Suomela E, Lehtovirta M, Kangas AJ, Jula A, et al. Metabolic profiling of fatty liver in young and middle-aged adults: Cross-sectional and prospective analyses of the Young Finns Study. Hepatology. 2017;65:491–500.

5. Preiss D, Rankin N, Welsh P, Holman RR, Kangas AJ, Soininen P, et al. Effect of metformin therapy on circulating amino acids in a randomized trial: the CAMERA study. Diabet Med. 2016;33:1569-1574
